# Evaluating the sensitivity of jurisdictional heterogeneity and jurisdictional mixing in national level HIV prevention analyses: context of the U.S. ending the HIV epidemic plan

**DOI:** 10.1186/s12874-022-01756-w

**Published:** 2022-11-26

**Authors:** Hanisha Tatapudi, Chaitra Gopalappa

**Affiliations:** 1 Department of Industrial and Management System Engineering, University of South Florida, Tampa, Florida USA; 2 Mechanical and Industrial Engineering, University of Massachusetts Amherst, Amherst, MA USA

**Keywords:** Jurisdictional mixing, Jurisdictional heterogeneity, HIV/AIDS, HIV intervention analysis, *Ending the HIV epidemic* (EHE) plan, Dynamic compartmental model, Simulation

## Abstract

**Background:**

The U.S. Ending the HIV epidemic (EHE) plan aims to reduce annual HIV incidence by 90% by 2030, by first focusing interventions on 57 regions (EHE jurisdictions) that contributed to more than 50% of annual HIV diagnoses. Mathematical models that project HIV incidence evaluate the impact of interventions and inform intervention decisions. However, current models are either national level, which do not consider jurisdictional heterogeneity, or independent jurisdiction-specific, which do not consider cross jurisdictional interactions. Data suggests that a significant proportion of persons have sexual partnerships outside their own jurisdiction. However, the sensitivity of these jurisdictional interactions on model outcomes and intervention decisions hasn’t been studied.

**Methods:**

We developed an ordinary differential equations based compartmental model to generate national-level projections of HIV in the U.S., through dynamic simulations of 96 epidemiological sub-models representing 54 EHE and 42 non-EHE jurisdictions. A Bernoulli equation modeled HIV-transmissions using a mixing matrix to simulate sexual partnerships within and outside jurisdictions. To evaluate sensitivity of jurisdictional interactions on model outputs, we analyzed 16 scenarios, combinations of a) proportion of sexual partnerships mixing outside jurisdiction: no-mixing, low-level-mixing-within-state, high-level-mixing-within-state, or high-level-mixing-within-and-outside-state; b) jurisdictional heterogeneity in care and demographics: homogenous or heterogeneous; and c) intervention assumptions for 2019–2030: baseline or EHE-plan (diagnose, treat, and prevent).

**Results:**

Change in incidence in mixing compared to no-mixing scenarios varied by EHE and non-EHE jurisdictions and aggregation-level. When assuming jurisdictional heterogeneity and baseline-intervention, the change in aggregated incidence ranged from − 2 to 0% for EHE and 5 to 21% for non-EHE, but within each jurisdiction it ranged from − 31 to 46% for EHE and − 18 to 109% for non-EHE. Thus, incidence estimates were sensitive to jurisdictional mixing more at the jurisdictional level. As a result, jurisdiction-specific HIV-testing intervals inferred from the model to achieve the EHE-plan were also sensitive, e.g., when no-mixing scenarios suggested testing every 1 year (or 3 years), the three mixing-levels suggested testing every 0.8 to 1.2 years, 0.6 to 1.5 years, and 0.6 to 1.5 years, respectively (or 2.6 to 3.5 years, 2 to 4.8 years, and 2.2 to 4.1 years, respectively). Similar patterns were observed when assuming jurisdictional homogeneity, however, change in incidence in mixing compared to no-mixing scenarios were high even in aggregated incidence.

**Conclusions:**

Accounting jurisdictional mixing and heterogeneity could improve model-based analyses.

**Supplementary Information:**

The online version contains supplementary material available at 10.1186/s12874-022-01756-w.

## Introduction

In the United States (U.S.), there were an estimated 1.18 million people living with HIV (PWH) as of 2019, and an estimated average of 36,500 new infections each year between 2015 and 2019 [1]. Although HIV disease has no cure, consistent use of antiretroviral therapy treatment (ART) by infected persons can fully suppress viral load, thus preventing transmissions [2]. Further, pre-exposure prophylaxis (PrEP) for high-risk susceptible individuals can reduce HIV acquisition by 99% [3]. However, there are considerable gaps in administering these preventive tools. As of 2019, nationally, 87% of PWH were aware of their infection (proportion aware), but only 66% of those aware were on ART with viral load suppression (proportion with VLS) [4]. Among susceptible persons with PrEP eligibility, only 23% were administered PrEP (PrEP coverage). In addition, there is considerable heterogeneity in these care proportions by age groups, risk groups, and jurisdictions [4]. Across geographical jurisdictions in the U.S., the proportion aware ranged from 80 to 96%, the proportion with VLS ranged from 49 to 83%, and PrEP coverage ranged from 6 to 93% [4].

Taking the above jurisdictional disparities into consideration, the most recent U.S. national strategic plan, Ending the HIV Epidemic (EHE) [5, 6], in addition to continuing the age and risk group focused efforts as in the previous national plan [7], also aims for jurisdictional focused efforts as follows. It aims to reduce national incidence by 75% by 2025 by focusing prevention efforts in 50 counties and 7 states (we will refer to these as the EHE jurisdictions), which had accounted for more than 50% of nationwide diagnoses in 2017, and reduce incidence by 90% by 2030 by expanding prevention efforts to the rest of the nation (we will refer to these as the non-EHE jurisdictions) [5].

Mathematical models that simulate future HIV incidence projections help evaluate the impact of interventions and inform intervention decisions. Recent literature includes multiple jurisdiction-specific models [8–16] and national level models [17, 18] that have conducted intervention analyses related to the U.S. EHE plan. However, there are certain gaps in these analyses. The jurisdiction-specific models evaluate each jurisdiction independently, which ignores the interactions between jurisdictions, specifically, the sexual partnership mixing between persons of different jurisdictions [19–24]. Also, they only focus on a small number of jurisdictions. On the other hand, the national level models do not consider the jurisdictional heterogeneity in population demographics, including population size of key risk groups such as injecting drug users and men who have sex with men (MSM) [25], and care parameters noted above, or the interactions between jurisdictions. Jurisdictional heterogeneity in demographics and care along with partnership mixing between jurisdictions suggest that there is potential for strategies adopted in one jurisdiction to influence the nation-wide HIV incidence [19–24]. However, the influence of these jurisdictional interactions, in light of the EHE plan, has not been studied, and there are certain gaps preventing such analyses. A model than can conduct such analyses is not available, and care data specific to jurisdiction and partnership mixing for every jurisdictional pair are also not fully available. Moreover, it is not clear if jurisdictional interactions influence epidemic projections (such as incidence and prevalence), or decisions inferred through models, or both.

To address these gaps in the literature, we developed a national HIV epidemic compartmental simulation model representative of the U.S. population and composed of 96 jurisdictions. The model’s construction enables us to evaluate the national epidemic as a whole, with geographical heterogeneity in population demographics, HIV epidemic, and interventions, and thus help identify what jurisdiction-specific strategies to adopt, such as how often to test, what should be the aim for retention-in-care, and what should be the target for PrEP coverage to achieve the intended goals of the EHE plan.

Using the model, we evaluated the sensitivity of jurisdictional mixing and jurisdictional heterogeneity on outcomes such as incidence, prevalence, and intervention decisions. Using the model’s comprehensive structure, we evaluated these metrics at different geographical levels, including individual jurisdictions, aggregated EHE jurisdictions, aggregated non-EHE jurisdictions, and national level aggregation. Understanding the significance of jurisdictional heterogeneity and mixing through these sensitivity analyses would inform data collection to ensure optimal use of resources, and subsequently inform model development based on the study’s objective and design. Note that the scope of work in this paper is limited to evaluating the sensitivity of jurisdictional interactions, under the intended goals of the model noted above, and not to infer decisions. However, upon availability of relevant data, the model can be easily updated to serve as a decision-analytic tool.

## Methods

### Compartmental model

**Fig. 1:**
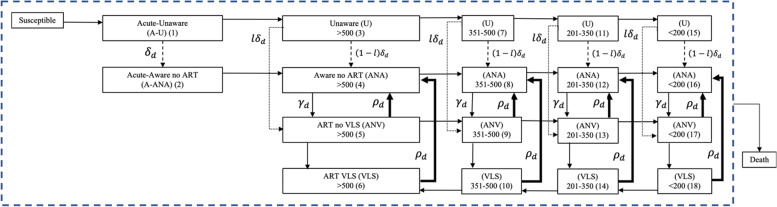
Compartmental simulation: Transition diagram with care continuum and disease stages. *δ*_*d*_: diagnostic rate in disease stage *d*, *l*: proportion linked to care within three months of diagnosis, *ρ*: care drop-out rate, and *γ*: rate of re-entry to care. Diagnostic rates for people linked to care at diagnosis (*lδ*_*d*_) (rates on  ; dashed arrow), diagnostic rates for people linked to care greater than 3 months after diagnosis ((1 − *l*)*δ*_*d*_) (rates on  ; dotted arrow), and care-drop-out rates (*ρ*_*d*_)(rates on  ; thick arrow) are estimated in the model specific to the scenario simulated. Rates corresponding to all other arrows are taken from data in literature and are presented in Table S2 of Appendix. (#): numbers in each compartment are used for referencing state transitions in Table S2

To simulate national-level HIV projections in the U.S., we developed two compartmental models, a National-Model and a Jurisdictional-Model. The compartmental stratifications in the National-Model included three sexual risk groups (HF, HM, MSM), eighty-eight age groups (individual ages from 13 to 100), four care continuum stages (Unaware, Aware no ART, ART no VLS, ART VLS), and five disease progression stages (Acute, CD4 > 500, CD4 350–500, CD4 200–350, CD4 <  200). By further stratifying the compartmental model into 96 geographical jurisdictions, the Jurisdictional-Model generated national-level HIV projections through simulation of the 96 epidemiological sub-models. We developed the models in Python programming. The flow diagram for care continuum transitions and disease stage progressions is depicted in Fig. 1. We only simulated sexually transmitted cases of HIV and did not model transmissions through injecting drug use. Below, prior to discussing each model in detail, we provide an overview of the mathematical formulation of the compartmental models.

Mathematically, a compartmental model to simulate an epidemic trajectory can be represented as a non-stationary continuous-time Markov process { *X*_*t*_ : Ω, *G*_*t*_, π_t_; *t* = 1 : *T*}, *X*_*t*_ being the state of an individual transitioning through states of an underlying Markov chain defined over a state space Ω, state-transitions governed by rates in a time-variant generator matrix *G*_*t*_, and the state distribution for the proportion of people in each state given by π_t_, for time *t* [26]. Here, we can represent the state space of the underlying Markov chain as Ω_nat_ = {[*R*, *A*, *S*, *C*, *D*], Death} for the National-Model and as Ω_jur_ = {[*R*, *A*, *S*, *C*, *D*, *J*], *Death*} for the Jurisdictional-Model, i.e., in addition to a ‘Death’ state, each of the other states is a multivariate state, where,

*R* represents risk-group, *R* ∈ {Heterosexual males, Heterosexual females, Men who have sex with men},

*A* represents age, *A* ∈ {13, 14, …, 100},

*S* represents infection status, *S* ∈ {Susceptible, Infected},

*C* represents HIV care status, {*C* ∈ {∅} *if S* = Susceptible; *C*∈ {Unaware, Aware no ART, ART no VLS, ART VLS} *if S* = Infected}, {∅} being the null set,

*D* represents HIV disease stage, {*D* ∈ {∅} *if S* = Susceptible, D ∈ {Acute, CD4 > 500, CD4 350–500, CD4 200–350, CD4 <  200} *if S* = Infected}, and

*J* represents geographical jurisdiction, *J* ∈ {1, 2, …, 96}.

Thus, the number of compartments in the National-Model is the size of its state space given by |Ω_nat_| = (3 × 88 × 1 × 1 × 1) + (3 × 88 × 1 × 4 × 5) + (1), where the first two elements represent the size (*R* × *A* × *S* × *C* × *D*), the first element (3 × 88 × 1 × 1 × 1) is the number of states corresponding to *S*= Susceptible, the second element (3 × 88 × 1 × 4 × 5) is the number of states corresponding to *S*= Infected, and the third element (1) corresponding to one Death state. Similarly, the number of compartments in the Jurisdictional-Model is the size of its state space given by |Ω_jur_| = (3 × 88 × 1 × 1 × 1 × 96) + (3 × 88 × 1 × 4 × 5 × 96) + (1) where the first two elements represent the size (*R* × *A* × *S* × *C* × *D* × *J*), the first element (3 × 88 × 1 × 1 × 1 × 96) is the number of states corresponding to *S*= Susceptible, the second element (3 × 88 × 1 × 4 × 5 × 96) is the number of states corresponding to *S*= Infected, and the third element (1) corresponding to one Death state.

The generator matrix *G*_*t*_ is a square matrix of size equal to the number of states, each element representing the monthly rates of transition between states. The rates of transitioning from any state with *S*= Susceptible to any state with *S*= Infected are estimated using a Bernoulli transmission equation (discussed below and formulated in Appendix Section A3). The rates for all other state-transitions, including states that transition persons across care stages, disease progression stages, aging, and mortality are based on data from the literature (discussed in more detail below, under description of each model, and the data are presented in Appendix Tables A2, A3, and A4). We assume persons do not transition across risk groups (i.e., *R*) or across jurisdictions (i.e., *J*), and thus rates corresponding to those state transitions are zero.

For this continuous-time representation, the solution to the state distribution (*π*_*t*_), which is an array of size |Ω_nat_| for the National-Model and |Ω_juri_| for the Jurisdictional-Model, with each element representing the proportion of people in that state at time *t*, is through solving for ${\pi}_t={\pi}_0{e}^{G_tt}$, using some initial state-distribution *π*_0_. However, considering the complexity of the model, specifically the time-variance of the generator matrix, solving it as such for continuous-time *t* is infeasible. Therefore, as typically done in compartmental model, we numerically solved it using ordinary differential equations (ODE) as per Euler’s approximation, starting at *π*_0_ and iteratively solving it at sufficiently small increments of discrete time-step ∆*t* (here monthly) as,

1 $${\pi}_{t+\Delta t}={\pi}_t+{\pi}_t{G}_t\Delta t$$

Further, in the Markov processes representation of the compartmental model, each element of *π*_*t*_ denotes the proportion of the population in that state, and thus, the sum of the elements add to 1. On the other hand, in the ODE representation of the compartmental model, without loss of information, we can denote each element of *π*_*t*_ as the number of people in that state, and thus, the sum of the elements add to the total population. Here, to determine the initial state distribution *π*_0_, we used data from the U.S. population (taking data from the U.S. Census Bureau for natural demographics related state distributions and data from the U.S. National HIV Surveillance Systems for determining HIV-related state distributions) in 2010 year-end for the National-Model and 2017 year-end for the Jurisdictional-Model. We then simulated *π*_*t*_ over calendar-time *t* using (1). Thus *π*_*t*_ represents the cross-sectional distribution of the U.S. population in a specific calendar-time *t*. Below, we discuss data and estimation processes for the components of the compartmental model in (1), including initialization of the model (*π*_2010_ for National-Model and *π*_2017_ for the Jurisdictional Model) and transition rates (elements of the time-variant generator matrix), specific to the National-Model and Jurisdictional-Model.

1. National-Model: The main purpose of the National-Model was, first, for calibration of sexual behavioral parameters specific to risk group and age group, as data were more widely available at the national level, and second, as a comparison against the Jurisdictional-Model to evaluate the sensitivity of jurisdictional heterogeneity and mixing. We briefly discuss the model here and present the data used for model calibration in the Appendix Sections A2 - A5.
  We initialized the National-Model to the 2010 HIV epidemic, by using data from the NHSS [26] to initially distribute the population into the different age groups, risk groups, disease stage and care continuum compartments (π_2010_). We simulated the model for the period 2011 to 2018, using (1) .
  We estimated incidence using a Bernoulli transmission equation (see Appendix Section A3) set as a function of behavior, distribution of PWH by care continuum, disease stage, disease and care continuum stage specific HIV transmission risk, PrEP coverage among susceptible population, and HIV acquisition risk by PrEP status. For the distribution of PWH by care continuum and disease stage, we calculated these in the simulation as the number of PWH in the respective care continuum and disease stage, and tracked it in the simulation over time. For PrEP coverage among susceptible persons, we used data from the NHSS [26]. Note, PrEP coverage in the simulation was initiated in year 2017 as it was only recently introduced and utilization in prior years were minimal (in 2017, 2.8% of the MSM population were on PrEP whereas in 2015, 0.7% of the MSM population were on PrEP [27]). We assumed PrEP only for MSM as < 1% of heterosexuals were administered PrEP in 2017 [28]). For HIV transmission risk by care continuum and disease stage of HIV infected persons, and HIV acquisition risk by PrEP-status of susceptible persons, we used data from the literature (see data in Appendix Table A12). For behavioral data, related to risk group specific partnership mixing, number of partners, proportion of partnership type (main or casual), average number of sexual acts per partner, number and type (anal or vaginal) of sexual acts per partner, and condom use and effectiveness, we used data from the literature (see data in Appendix Tables A5-A11). For the remaining behavioral data related to per act probability of transmission, and partnership mixing by age group we used data ranges from the literature and calibrated the values by fitting simulated incidence to the surveillance estimates of national incidence over the period 2011 to 2018 (see data in Appendix Tables A13, A5, A6, and A9).
  For the rates of transitioning across disease stages (acute, and CD4-count stages) we used data from the literature (see Table A2 Appendix). For simulating the transitioning between the care continuum stages, we used data from the literature for rate of achieving VLS when on ART and for rate of re-entry-to-care after dropping out, and data from the NHSS for the proportion linking to care at diagnosis (see Table A2 in Appendix). The remaining two care continuum transition parameters, i.e., rates of HIV-diagnosis and care-drop-out, are dependent on testing and retention-in-care interventions, which are likely to change over time. Thus, we estimated these rates in the simulation by fitting to the annual NHSS data on care continuum distributions (see estimation method in Appendix Section A4).
2. Jurisdictional-Model: In this model, we further split the model into 96 jurisdictions. These jurisdictions represent 54 EHE jurisdictions (47 counties and 7 states) and 42 non-EHE jurisdictions (42 of the remaining 43 states) (Table A1 in Appendix). We did not model 3 of the 50 EHE counties stated in the EHE plan and 1 non-EHE state due to data unavailability (see Table A1 in Appendix). We initialized the Jurisdictional-Model to 2017 as jurisdiction-specific HIV related data were only available for 2017, 2018, and 2019 at the time of this study. To initially distribute the population into the different compartments, we used census data for the overall population sizes [29], NHSS data for HIV population by age group, risk group and care-continuum stage [26], and estimates from the literature for the proportion of MSM among adult males [25] (see data in Table A14 in Appendix), using jurisdiction-specific data when available. As data for care continuum distributions specific to risk group within each jurisdiction were not available, we assumed that the ratio of risk group specific metric to overall population metric observed at the national level would be the same as the ratio at the jurisdictional-level. Specifically, we applied this simplified assumption to two metrics within each jurisdiction, proportion aware and proportion with ART VLS (see Appendix Section A5). We normalized the values to ensure the sum of the proportions across all care continuum stages (Unaware, Aware no ART, ART no VLS, ART VLS) is equal to 1 for each jurisdiction. We did not model jurisdictions that did not have prevalence data, i.e., where data was either unavailable or suppressed (see Table A1 in Appendix for the list of jurisdictions modeled and excluded).
  We estimated incidence rates using a Bernoulli transmission equation as in the National-Model, i.e., as a function of behavior, distribution of PWH by care continuum and disease stage, disease and care continuum stage specific HIV transmission risk, PrEP coverage among susceptible population, and HIV acquisition risk by PrEP status. For behavioral data, under the assumption that sexual behavior only changes by risk and age group, and not geography, we used the same data (including the calibrated values) as in the National-Model for every jurisdiction. Additionally, we modeled interactions between jurisdictions by using a jurisdictional mixing matrix for partnership formation, using data from behavioral surveys and phylogenetic studies [19–24], and evaluating scenarios with varying levels of mixing to test its sensitivity. For the distribution of PWH by care continuum and disease stage, as with the National-Model, we calculated these in the simulation, but specific to jurisdiction, as the number of PWH in care continuum and disease stage are tracked in the simulation over time. For PrEP coverage among susceptible persons, as with the National-Model we used data from the NHSS, except to evaluate the sensitivity of jurisdictional variations in access to PrEP, we implemented scenarios that used the national PrEP coverage for every jurisdiction and jurisdiction-specific PrEP coverage. We discuss the scenarios under *Scenarios modeled*.
  The rates of progression across disease stages (acute, and CD4-count stages) are related to natural disease epidemiology, thus, we used the same literature data as in the National-Model. For simulating the transitioning between the care continuum stages, we used the same data as that in the National-Model for the rate of achieving VLS when on ART and for the rate of re-entry-to-care after dropping. We believe these are reasonable as, in our model, the former relates to the natural epidemiology, and the latter is not based on intervention and partially based on natural disease progression.
  The remaining two care continuum transition parameters, i.e., rates of HIV-diagnosis and care-drop-out, are related to testing and retention-in-care interventions, and thus, likely to vary across jurisdictions and over time. Thus, as with the National-Model, we estimated these rates in the simulation by fitting to the annual NHSS data on care continuum distributions. Additionally, to test the sensitivity of jurisdictional variations, we evaluated multiple scenarios. We evaluated scenarios that applied the same rates estimated by the National-Model to every jurisdiction (jurisdictional homogeneity). We also evaluated scenarios that estimated rates by fitting to jurisdiction-specific NHSS data on care continuum distributions (jurisdictional heterogeneity) [26] but using the same method as in the National-Model (described in Appendix Section A4). These scenarios are described in more detail under *Scenarios modeled.*
  To analyze the sensitivity of jurisdictional mixing and heterogeneity, we simulated the epidemic using the Jurisdictional-Model for the period 2018 to 2030, under two sets of intervention assumptions. One is a continuation of status-quo intervention (baseline). The second is the adoption of interventions as per the EHE strategy to meet EHE care targets (EHE plan). Specifically, the EHE adopts a strategy of diagnose (through increased testing), treat (through increased linkage to care and retention), and prevent (through PrEP), as three of its four strategic pillars. Its prevention efforts are aimed to reach care continuum targets of 95–95-95 (i.e., 95% awareness of infection status among PWH, 95% linkage to care among those aware, and 95% VLS among those in care) and PrEP coverage of 50% among those eligible for PrEP, by 2025 in EHE jurisdictions, and by 2030 in all jurisdictions nation-wide [30]. We discuss these scenarios modeled in more detail below.

### Scenarios modeled

We used the Jurisdictional-Model to simulate the HIV epidemic for the period 2018 to 2030. The time-unit in the model was monthly. We evaluated 16 scenarios to analyze the sensitivity of jurisdictional heterogeneity in care continuum and the sensitivity of jurisdictional mixing. Details of each scenario are discussed and summarized in Table 1, their broad differences are as follows. Scenarios S1 to S8 assumed homogeneity in care across jurisdictions by using national level estimates for HIV-diagnosis rate, care-drop-out rate, and PrEP coverage for every jurisdiction, and homogeneity in risk group distribution by assuming national level estimates for the proportion of the population who are MSM [25]. On the other hand, scenarios S9 to S16 assumed heterogeneity in care across jurisdictions by estimating jurisdiction-specific HIV-diagnosis rate, care-drop-out rate, and PrEP coverage, and heterogeneity in risk group distribution by using jurisdiction-specific estimates for proportion MSM [25]. Scenarios S1 to S4 and S9 to S12 assumed continuation of status-quo interventions by using baseline year (2018) estimates for HIV-diagnosis rate, care-drop-out rate, and PrEP coverage, and keeping it constant over the period 2019 to 2030. Scenarios S5 to S8 and S13 to S16 modeled the EHE plan by using time-varying values for HIV-diagnosis rate, care-drop-out rate, and PrEP coverage, estimated to reach the EHE targets (95–95-95 care targets and 50% PrEP coverage among eligible) by 2025 for EHE jurisdictions and by 2030 for non-EHE jurisdictions. Scenarios S1, S5, S9, and S13 assumed no-mixing between jurisdictions, Scenarios S2, S6, S10, and S14 assumed lower levels of mixing between jurisdictions within the same state but no-mixing outside state (Level-1-mixing), Scenarios S3, S7, S11, and S15 assumed higher levels of mixing between jurisdictions within the same state but no-mixing outside state (Level-2-mixing), and Scenarios S4, S8, S12, and S16 assumed higher levels of mixing between jurisdictions within the same state and mixing outside state (Level − 3-mixing).

**Table 1:** Scenarios simulated using the Jurisdictional-Model

***Scenario no.***	***Mixing assumption***^**‡**^	***Care Intervention*** ***(HIV-diagnosis rate, care-drop-out rate, and PrEP coverage)***		***Jurisdictional heterogeneity assumption***
		***EHE jurisdictions***	***non-EHE jurisdictions***	
**[S1]**	**No-mixing**	**Baseline** (2018): Values kept constant at 2018 national estimates for all years	**Baseline** (2018): Values kept constant at 2018 national estimates for all years	**Homogeneous** care and risk group distribution: national estimates used for all jurisdictions
**[S2]**	**Level 1-mixing**			
**[S3]**	**Level-2 mixing**			
**[S4]**	**Level-3 mixing**			
**[S5]**	**No-mixing**	**EHE plan**: Values calibrated to nationally achieve EHE targets (95–95-95) by 2025, and kept constant at 2025 value thereafter	**EHE plan**: Values kept constant at 2018 national estimates until 2025, and thereafter, calibrated to nationally achieve EHE targets (95–95-95) by 2030	
**[S6]**	**Level 1-mixing**			
**[S7]**	**Level-2 mixing**			
**[S8]**	**Level-3 mixing**			
**[S9]**	**No-mixing**	**Baseline** (2018): Values kept constant at 2018 jurisdiction-specific for all years	**Baseline** (2018): Values kept constant at 2018 jurisdiction-specific for all years	**Heterogeneous** care and risk group distribution: jurisdiction-specific estimates
**[S10]**	**Level 1-mixing**			
**[S11]**	**Level-2 mixing**			
**[S12]**	**Level-3 mixing**			
**[S13]**	**No-mixing**	**EHE plan**: Jurisdiction-specific estimates calibrated to achieve EHE targets (95–95-95) within each jurisdiction by 2025, and kept constant at 2025 value thereafter	**EHE plan**: Jurisdiction-specific estimates kept constant at 2018 values until 2025, and thereafter, calibrated to achieve EHE targets (95–95-95) by 2030 within each jurisdiction	
**[S14]**	**Level 1-mixing**			
**[S15]**	**Level-2 mixing**			
**[S16]**	**Level-3 mixing**			

^‡^See Table 2 for data assumptions in each mixing category

#### Jurisdictional mixing assumptions

As noted above, we evaluated no-mixing and three types of mixing scenarios. Data for partnership mixing between persons of different jurisdictions are limited, and national level survey data are unavailable. However, recent phylogenetic studies, which used nucleotide sequence data of persons with recent HIV diagnoses in the U.S., which infer close transmissions by creating pairwise links between persons with closely related viral DNA sequence, serve as a suitable source [24]. It must be noted that links formed through nucleotide sequencing do not necessarily indicate direct transmissions (i.e., direct partnership links) and are generated using data from only positive persons with recent diagnoses. However, as these studies are conducted at the national level, they serve as suitable reference points for informing the sensitivity analyses in our study.

To inform the range of values for our sensitivity analyses, we used the following sources, which included surveys and phylogenetic studies. Results from a survey of Baltimore heterosexual males suggest that almost 50% of the participants chose partners in the same or adjacent census tract [19]. Study presented by Gesink et al., conducted interviews with MSM from Toronto, Canada and reported that 30% of the study’s participants had sex with partners outside of their town [23]. Study of phylogenetic analysis of HIV sequences in Shanghai by Li et al., states that 33.8% of the HIV sequences analyzed were associated to infected individuals from another province [22]. A phylogenetic study by Oster et al., using data from young black MSM between ages 16 to 24 in Mississippi, found signifcant number of clusters with persons from more than one jurisdiction, suggesting significant mixing between jurisdictions [20]. A follow-up survey of young black MSM between ages 16 to 24 (by Oster et al.,) found that 20% of the persons reported travelling to another region in or outside the state of Mississippi, thus supporting observations in the phylogenetic study [20]. Phylogenetic study by Board et al. used nucleotide sequence data of persons with recent HIV diagnoses across the U.S. to identify proportion of pairs with closely related sequences that were between persons of different jurisdictions, which at the time of this study was the most comprehensive nationally representative study of the U.S population. This study also provides the most comprehensive information by presenting data specific to risk groups and jurisdiction types (within county, within state, and outside state). Data from this study showed that among risk groups, links ranged between 47 to 65% within the same county, between 78 to 88% within the same state, and the remaining between persons of different states [24]. Because links do not represent direct transmissions, they do not represent partnership links.

For data inferred through phylogenetic analyses it is infeasible to determine the time period of mixing. Most data reported from the above behavioral surveys were from partnerships reported over a period of 12 months [19–24]. Therefore, we used these data to model the proportion of annual partnerships that are with persons outside their jurisdiction. We used data from [24], which is the most comprehensive and nationally representative, to inform two sets of scenarios. Further, as mixing is likely to vary by jurisdiction and the reported proportions mixing outside jurisdiction in all the above studies were on the higher end, to test the sensitivity of this parameter, we evaluated one additional scenario using lower values of 90 to 85%, which is below the lowest observed in the behavioral survey studies [19–24]. The mixing assumptions are summarized in Table 2 and explained below.

1. No-mixing (S1, S5, S9, and S13 in Table 1): For these scenarios we assumed partnership mixing was 100% within jurisdiction, and 0% outside jurisdiction.
2. Level-1-mixing (S2, S6, S10, and S14 in Table 1): For these scenarios we assumed persons in a jurisdiction could have partnerships with persons in other jurisdictions but within the same state and not with persons in other states. If the jurisdiction modeled is an EHE county or a non-EHE state with EHE counties within it, we used the following for proportion mixing-within-jurisdiction: 90% for HM and HF, and 85% for MSM (Table 2). For the proportion mixing with the other jurisdictions within the state we used 1 minus mixing-within-jurisdiction. If there are multiple EHE counties within a state, we split the value (1 minus mixing-within-jurisdiction) equally between the other EHE jurisdictions and the rest of the state. If the jurisdiction modeled is a state with no EHE counties within it, we assumed 100% mix within their state.
3. Level-2-mixing (S3, S7, S11, and S15 in Table 1): These scenarios were modeled exactly as in Level-1-mixing, except that we assumed higher levels of outside mixing. Specifically, we used the following data from [24] for the proportion mixing-within-jurisdiction: 57% for HM, 65% for HF, and 47% for MSM (Table 2).
4. Level-3-mixing (S4, S8, S12, and S16 in Table 1): For these scenarios we assumed persons in a jurisdiction could have partnership with persons in any jurisdiction. If the jurisdiction modeled is an EHE county or a non-EHE state with EHE counties within it, we used the following data from [24] for the proportion mixing-within-jurisdiction: 57% for HM, 65% for HF, and 47% for MSM (Table 2). We used mixing-within-state minus mixing-within-jurisdiction data for the proportion mixing within state but outside their own jurisdiction (28% for HM, 23% for HF, and 31% for MSM), and distributed it equally among the jurisdictions within state. We used 1 minus mixing-within-state for mixing-outside-state and distributed it across all other states weighted by distance to the state. If the jurisdiction modeled is a state without EHE counties within it, we used mixing-within-state data from [24] for mixing within jurisdiction and distributed the remaining across all other states weighting by the distance to that state. We used the Euclidean distance between the geographical co-ordinates (latitude and longitude) of two states as a proxy for the distance between jurisdictions.

**Table 2:** Assumptions of sexual partnership mixing across jurisdictions

***Mixing assumption ➔***	Level-1mixing^**a**^			Level-2 mixing^**a**^			Level-3 mixing^**a**^		
***Risk group ➔***	HM	HF	MSM	HM	HF	MSM	HM	HF	MSM
***Jurisdictional interaction category***									
**Same jurisdiction**	90%	90%	85%	57%	65%	47%	57%	65%	47%
**Other jurisdiction same state**	10%	10%	15%	43%	35%	53%	28%	23%	31%
**Other states**	0%	0%	0%	0%	0%	0%	14%	12%	22%

*HM* Heterosexual males, *HF* Heterosexual females, *MSM* Men who have sex with men;

^a^Values represent proportion of partnership mixing with the jurisdictional interaction category

#### Evaluating sensitivity of jurisdictional mixing while keeping jurisdictional homogeneity in care

We used the Jurisdictional-Model to evaluate the sensitivity of jurisdictional mixing when assuming jurisdictional homogeneity in care. Both when keeping interventions at baseline, i.e., HIV-diagnosis rate, care-drop-out rate, and PrEP coverage constant over the period 2018 to 2030 at 2018 baseline values (S1, S2, S3, and S4), and when scaling-up interventions over time to meet the 95–95-95 care and 50% PrEP targets (S5, S6, S7, and S8). To model jurisdictional homogeneity in care, we initialized the care continuum distribution of each jurisdiction to be equal to the national level for year 2017 year-end. We explain these scenarios in more detail below.

1. **Baseline-intervention; jurisdictional homogeneity in care (S1, S2, S3, and S4 in Table** 1): In these scenarios, we used the baseline estimates derived by the National-model for rates of HIV-diagnosis and care-drop-out, fitted to the national care continuum distribution in 2018. We kept these rates constant for the following years (i.e., 2019 to 2030) and used the same rates for all jurisdictions. We also kept PrEP-coverage constant at 2018 national level for all years and all jurisdictions. While we used jurisdiction-specific data for PWH in each risk group, we assumed that the proportion of the population who are MSM is the same for every jurisdiction and used national level estimates from [25].
2. **EHE-plan-intervention; jurisdictional homogeneity in care (S5, S6, S7, and S8 in Table** 1): In these scenarios, we used national level estimates for the scale-up in interventions (HIV-diagnosis rate, care-drop-out rate, and PrEP coverage) to meet the EHE targets. Specifically, we used the National-Model to estimate the HIV-diagnosis rate, care-drop-out rate, and PrEP coverage necessary to linearly scale-up care continuum proportions and PrEP coverage from its national baseline values in year 2018 to the EHE targets. For EHE jurisdictions, the interventions were linearly scaled over the period 2019 to 2025 and kept constant thereafter, and for the non-EHE jurisdictions, the values were kept constant for the period 2018 to 2025 and linearly scaled-up over the period 2026 to 2030. To recollect, the EHE targets were 95–95-95 for the care continuum and 50% for PrEP coverage among those eligible. In the U.S., PrEP eligibility is based on specific indicators, such as a person’s risk factor for acquiring HIV and recency in other sexually transmitted infections [31]. In the model, we do not simulate these PrEP indicators, thus, we use the reported number for persons with PrEP indicators to determine the percentage of susceptible population who are eligible for PrEP and take 50% of that percentage as the EHE target for PrEP coverage. Thus, a 50% coverage among those eligible would approximately be equal to 15% coverage among all susceptible. Not modeling PrEP indicators but using the above conversion is equivalent to assuming that sexual behaviors do not change over time, which is reasonable for the scope of our analyses as we do not evaluate behavioral interventions.

#### Evaluating sensitivity of jurisdictional mixing and jurisdictional heterogeneity in care

We used the Jurisdictional-Model to evaluate the sensitivity of jurisdictional mixing and jurisdictional heterogeneity in care. Both when keeping HIV-diagnosis rate, care-drop-out rate, and PrEP coverage constant over the period 2018 to 2030 (Scenarios S9, S10, S11, and S12), and when scaling-up over time to meet the 95–95-95 targets (Scenarios S13, S14, S15, and S16). To model jurisdictional heterogeneity in care, we initialized the model to jurisdiction-specific care data for 2017 year-end and estimated jurisdiction-specific HIV-diagnosis rates, care-drop-out rates, and PrEP-coverage for the period 2019 to 2030. We explain these scenarios in more detail below.

1. **Baseline-intervention; jurisdiction-heterogeneity in care (S9, S10, S11, and S12 in** **Table** 1**)**: In these scenarios, we used the jurisdiction-specific estimates for HIV-diagnosis rate, care-drop-out rate, and PrEP coverage. We derived jurisdiction-specific HIV-diagnosis rate and care-drop-out rate in the Jurisdictional-Model by using the 2018 jurisdiction-specific care continuum distributions and kept it constant for the period 2019 to 2030. We also used 2018 jurisdiction-specific estimates of PrEP coverage and kept it constant for the period 2019 to 2030.
2. **EHE-plan-intervention; jurisdiction-heterogeneity in care (S13, S14, S15, and S16 in** **Table** 1**)**: In these scenarios, we used jurisdiction-specific estimates for the scale-up in interventions (HIV-diagnosis rate, care-drop-out rate, and PrEP coverage) to meet the EHE targets. Specifically, we used the Jurisdictional-Model to estimate jurisdiction-specific HIV-diagnosis rate, care-drop-out rate, and PrEP coverage necessary to linearly scale-up care continuum proportions and PrEP coverage from its jurisdiction-specific baseline values in year 2018 to the EHE target values and years. That is, for EHE jurisdictions, the interventions were linearly scaled over the period 2019 to 2025 and kept constant thereafter, and for the non-EHE jurisdictions, the values were kept constant for the period 2019 to 2025 and linearly scaled-up over the period 2026 to 2030. To recollect, the EHE targets were 95–95-95 for the care continuum and 50% for PrEP coverage among those eligible. PrEP eligibility was determined in the same manner as described above for EHE-plan-intervention; jurisdictional homogeneity scenarios (S5, S6, S7, and S8).

### Model verification and output metrics

**Fig. 2:**
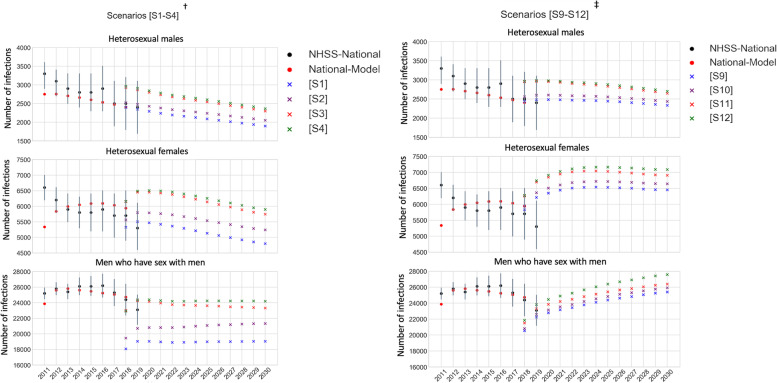
Comparing annual risk group specific incidence projections between NHSS, National-Model, and Jurisdictional-Model^*^. NHSS: National HIV Surveillance System; NHSS-National: national level estimates from NHSS; ^*^Using period 2011 to 2019 to validate that National-Model simulated estimates are within range of NHSS estimates; using period 2018 to 2019 to verify that Jurisdictional-Model simulated estimates (from scenarios S1 to S4 ^†^, and S9 to S12^‡^) generate incidence in magnitudes similar to National-Model estimates; and using period 2020 to 2030 to observe differences between Jurisdictional-Model scenarios S1, S2, S3, S4, S9, S10, S11, and S12. ^†^ Scenarios S5, S6, S7, and S8, start at the same baseline (2018) as S1, S2, S3, and S4, respectively. ^‡^ Scenarios S13, S14, S15, and S16, start at the same baseline (2018) as S9, S10, S11, and S12, respectively

As the sexual behavioral parameters in the National-Model were calibrated to the national incidence between 2011 and 2018, and because these parameters were then used in the Jurisdictional-Model, we first verified that the annual risk group specific incidence simulated by the National-Model compares well with NHSS estimates for years 2011 to 2018 (Fig. 2).

Results suggest overall good fit to NHSS incidence for all three risk groups.

For each of the 16 scenarios simulated in the Jurisdictional-Model, and for each jurisdiction, we extract the following metrics for the period 2018 to 2030: incidence as number of new infections per year, prevalence as the total number of PWH in that year, HIV-testing interval as the inverse of the HIV-diagnosis rate (a proxy), and retention-in-care rates as 1 minus the care-drop-out rate. HIV-testing intervals and retention-in-care rates serve as decision metrics to inform HIV testing and retention-in-care intervention programs, and incidence and prevalence projections serve as expected outcomes from implementing those decisions. We compare these metrics across the 16 scenarios to infer the sensitivity of model outputs to jurisdictional mixing and jurisdictional heterogeneity in care.

## Results

**Fig. 3:**
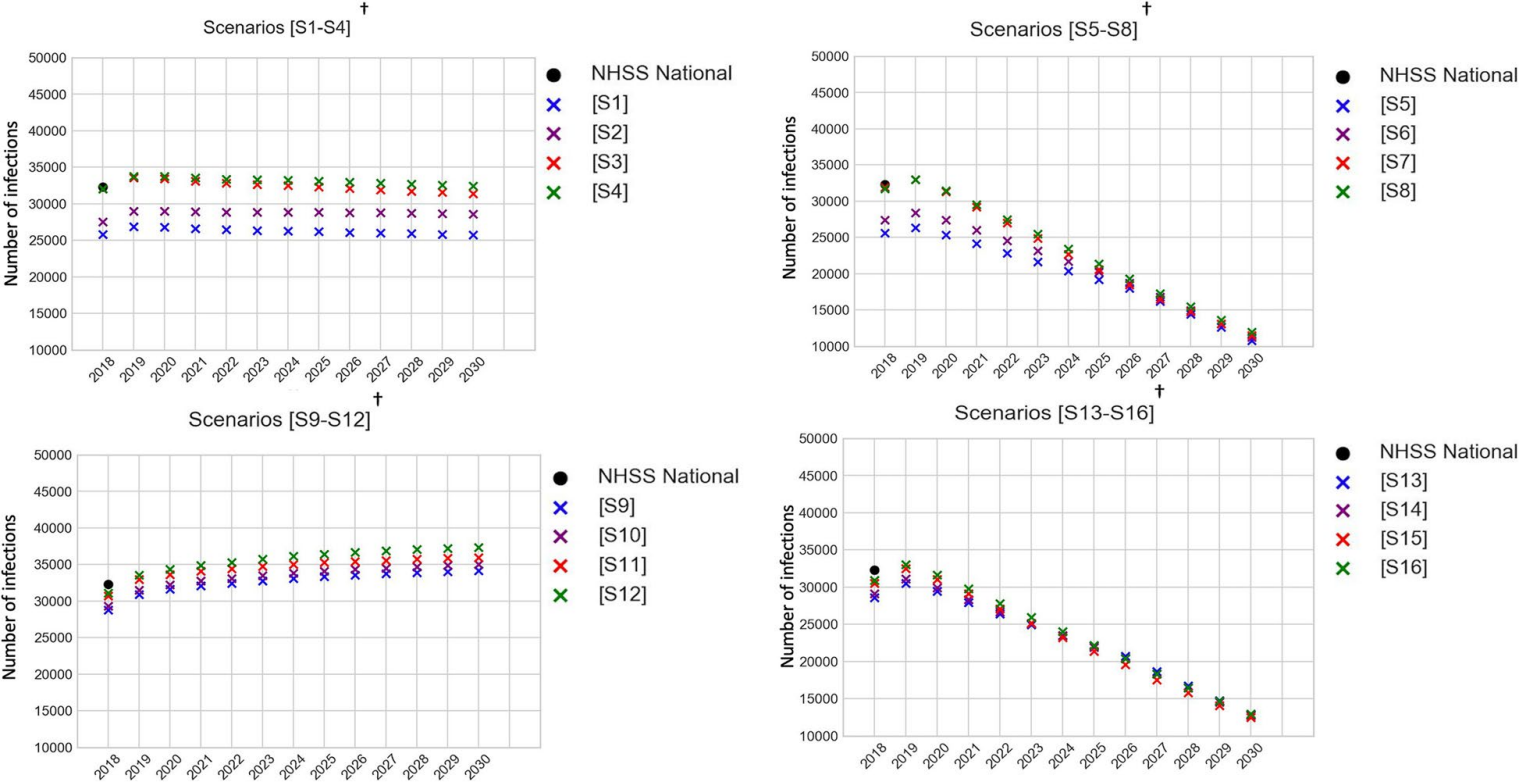
Comparing annual incidence projections between 16 scenarios simulated in Jurisdictional-Model and NHSS estimates^*^. NHSS: National HIV Surveillance System; NHSS-National: national level estimates from NHSS. ^*^Using period 2018 to 2019 to verify that Jurisdictional-Model simulated estimates generate incidence in magnitudes similar to NHSS estimates; and using period 2020 to 2030 to observe differences between Jurisdictional-Model scenarios S1 to S16. ^†^Scenarios S1 to S8 assume jurisdictional homogeneity; Scenarios S9 to S16 assume jurisdictional heterogeneity; Scenarios S1 to S4 and S9 to S12 assume baseline intervention; Scenarios S5 to S8 and S13 to S16 scale-up interventions as per EHE-plan

While the risk-specific incidence estimates from the National-Model were within the range of NHSS estimates over the period 2011 to 2018 (as mentioned in Model Verification), as expected from the design of the scenarios, the fit of incidence estimates from the Jurisdictional-Model varied by assumptions in jurisdictional mixing and heterogeneity (Fig. 2). Specifically, for years 2018 and 2019, risk group specific incidence (Fig. 2) and total incidence (Fig. 3) estimated by the Jurisdictional-Model were sensitive to jurisdictional mixing (comparing no-mixing scenarios S1, S5, S9 and S13, with Level-1-mixing scenarios S2, S6, S10, and S14, Level-2-mixing scenarios S3, S7, S11, and S15, and Level-3-mixing scenarios S4, S8, S12, and S16).

We did not attempt to calibrate behavioral data to improve the fit for each scenario as our objective is to test the sensitivity of jurisdictional mixing and heterogeneity in care and demographics while keeping all else fixed. Further, the Jurisdictional-Model excluded some counties and states due to data suppression from small data. However, the magnitude of the estimates are close to the national ranges, providing verification that the Jurisdictional-Model, which simulated local HIV epidemics in 96 jurisdictions can collectively generate results close to the overall national estimates.

In baseline-intervention scenarios, incidence projections for the period 2018 to 2030 were sensitive to jurisdictional mixing, both when assuming jurisdictional homogeneity in care (S1 compared to S2, S3 and S4) and jurisdictional heterogeneity in care (S9 compared to S10, S11, and S12) (Fig. 2), but more so in the former than the latter as seen by the percent change in incidence (Table 3). Compared to S9, the aggregated national incidence in S10, S11, and S12 changed by 2 to 2%, 7 to 5%, and 8 to 9%, respectively, whereas, compared to S1, the aggregated national incidence in S2, S3, and S4 changed by 7 to 11%, 24 to 22%, and 24 to 26%, respectively, the range corresponding to years 2018 to 2030 (see ‘All’ risk group ‘National’ in Table 3).

**Table 3:** Change in aggregated incidence^a^ (2018, 2030)^b^ in mixing scenarios compared to no-mixing (baseline intervention^c^)

***Scenario no. ➔***		S1	S2	S3	S4	S9	S10	S11	S12
***Care assumption ➔***		Homogeneity in care across jurisdictions (2018, 2030)^b^				Heterogeneity in care across jurisdictions (2018, 2030)^b^			
***Mixing assumption ➔***		No-mixing	Mixing level 1	Mixing level 2	Mixing level 3	No-mixing	Mixing level 1	Mixing level 2	Mixing level 3
***Risk group***	***Jurisdiction*** ***type***								
**All**	**National**	Ref	7, 11%	24, 22%	24, 26%	Ref	2, 2%	7, 5%	8, 9%
	**EHE**	Ref	-3, −4%	−10, −8%	−5, − 4%	Ref	−1, −1%	−2, −2%	0, 0%
	**Non-EHE**	Ref	19, 28%	67, 55%	60, 60%	Ref	5, 7%	18, 15%	19, 21%
**HM**	**National**	Ref	5, 8%	22, 21%	24, 25%	Ref	5, 4%	21, 14%	21, 16%
	**EHE**	Ref	−2, −4%	−9, −10%	0, − 4%	Ref	−2, − 2%	−8, −7%	−6, −6%
	**Non-EHE**	Ref	15, 25%	65, 65%	57, 64%	Ref	14, 11%	59, 36%	57, 39%
**HF**	**National**	Ref	4, 9%	15, 20%	16, 23%	Ref	2, 3%	7, 7%	8, 10%
	**EHE**	Ref	−2, −4%	−8, −9%	−5, − 5%	Ref	− 1, − 1%	−3, −3%	−2, −3%
	**Non-EHE**	Ref	14, 26%	50, 56%	45, 59%	Ref	5, 7%	17, 16%	17, 21%
**MSM**	**National**	Ref	8, 12%	27, 22%	27, 27%	Ref	1, 2%	5, 4%	6, 9%
	**EHE**	Ref	−3, −4%	−11, −8%	−5, − 4%	Ref	0, − 1%	− 1, −2%	1, 2%
	**Non-EHE**	Ref	20, 29%	72, 54%	64, 60%	Ref	4, 6%	14, 12%	15, 19%

*HM* Heterosexual males, *HF* Heterosexual females, *MSM* Men who have sex with men

National: aggregate of all EHE and non-EHE jurisdictions; EHE: aggregate of all EHE jurisdictions; Non-EHE: aggregate of all non-EHE jurisdictions

^a^ % change in incidence in mixing compared to no-mixing scenario = 100 × (mixing scenario – no-mixing scenario)/mixing scenario)

^b^ Values presented are for years 2018, 2030, respectively, and represents the range over the duration of the simulation

^c^ Scenarios 1 to 4 and 9 to 12 keep care metrics (HIV-diagnosis rate, care-drop-out rate, and PrEP coverage) fixed at the 2018 baseline values for the full duration of the simulation

In EHE-plan-intervention scenarios, in 2018, the percent change in incidence in mixing compared to no-mixing were similar to that in baseline-intervention scenarios above, which is expected as they start at the same baseline in 2018. However, as incidence decreased over the period 2019 to 2030 from scale-up of care, the differences diminished (Fig. 3, Table 4). Compared to S13, the aggregated national incidence in S14, S15, and S16 changed by 2% to − 1, 7% to − 3, and 8% to 1%, respectively, and compared to S5, the aggregated national incidence in S6, S7, and S8 changed by 7 to 4%, 24 to 6%, and 24 to 10%, respectively, the range corresponding to years 2018 and 2030 (see ‘All’ risk group ‘National’ in Table 4). While the care metrics in S13 to S16 were estimated in the Jurisdictional-model during the simulation and thus varied by scenario and jurisdiction, the care metrics in S5 to S8 were extracted from the National-model and thus were constant across scenarios and jurisdictions. Therefore, diminishing differences in both sets of scenarios suggest that, while incidence is sensitive to jurisdictional mixing when incidence was high, as incidence decreases, the sensitivity of mixing diminishes.

**Table 4:** Change in aggregated incidence^a^ (2018, 2030)^b^ in mixing scenarios compared to no-mixing (EHE plan^c^)

***Scenario no. ➔***		S5	S6	S7	S8	S13	S14	S15	S16
***Care assumption ➔***		Homogeneity in care across jurisdictions (2018, 2030)^**b**^				Heterogeneity in care across jurisdictions (2018, 2030)^**b**^			
***Mixing assumption ➔***		No-mixing	Mixing level 1	Mixing level 2	Mixing level 3	No-mixing	Mixing level 1	Mixing level 2	Mixing level 3
***Risk group***	***Jurisdiction *** ***type***								
**All**	**National**	Ref	7, 4%	24, 6%	24, 10%	Ref	2, −1%	7, −3%	8, 1%
	**EHE**	Ref	−3, 0%	−10, 1%	−5, 7%	Ref	− 1, 4%	−2, 8%	0, 15%
	**Non-EHE**	Ref	19, 7%	67, 9%	60, 13%	Ref	5, −4%	18, − 11%	19, − 9%
**HM**	**National**	Ref	5, 4%	22, 10%	23, 13%	Ref	5, 0%	21, 0%	21, 5%
	**EHE**	Ref	−2, −2%	−9, −4%	−6, 0%	Ref	−2, 0%	−8%, − 25	−6, 2%
	**Non-EHE**	Ref	15, 9%	65, 21%	63, 24%	Ref	14, 0%	59, 5%	57, 6%
**HF**	**National**	Ref	4, 4%	15, 7%	15, 10%	Ref	2, −2%	7, −5%	8, − 2%
	**EHE**	Ref	−2, −1%	−8, 0%	−7, 4%	Ref	− 1, 2%	−3, 4%	− 2, 8%
	**Non-EHE**	Ref	14, 7%	50, 11%	47, 14%	Ref	5, −3%	17, − 9%	17, − 7%
**MSM**	**National**	Ref	8, 4%	28, 6%	26, 10%	Ref	1, − 1%	5, − 3%	6, 2%
	**EHE**	Ref	−3, 0%	− 11, 2%	− 9, 8%	Ref	0, 5%	− 1, 10%	1, 18%
	**Non-EHE**	Ref	20, 6%	72, 8%	67, 11%	Ref	4, − 5%	14, − 13%	15, − 11%

*HM* Heterosexual males, *HF* Heterosexual females, *MSM* Men who have sex with men

National: aggregate of all EHE and non-EHE jurisdictions; EHE: aggregate of all EHE jurisdictions; Non-EHE: aggregate of all non-EHE jurisdictions;

^a^ % change in incidence in mixing compared to no-mixing scenario = 100 × (mixing scenario – no-mixing scenario)/mixing scenario)

^b^ Values presented are for years 2018, 2030, respectively, and represents the range over the duration of the simulation

^c^ Scenarios 5 to 8 and 13 to 16 scale-up care metrics (HIV-diagnosis rate, care-drop-out rate, and PrEP coverage) from 2018 baseline to reach EHE targets by 2025 for EHE jurisdictions and by 2030 for non-EHE jurisdictions

**Fig. 4:**
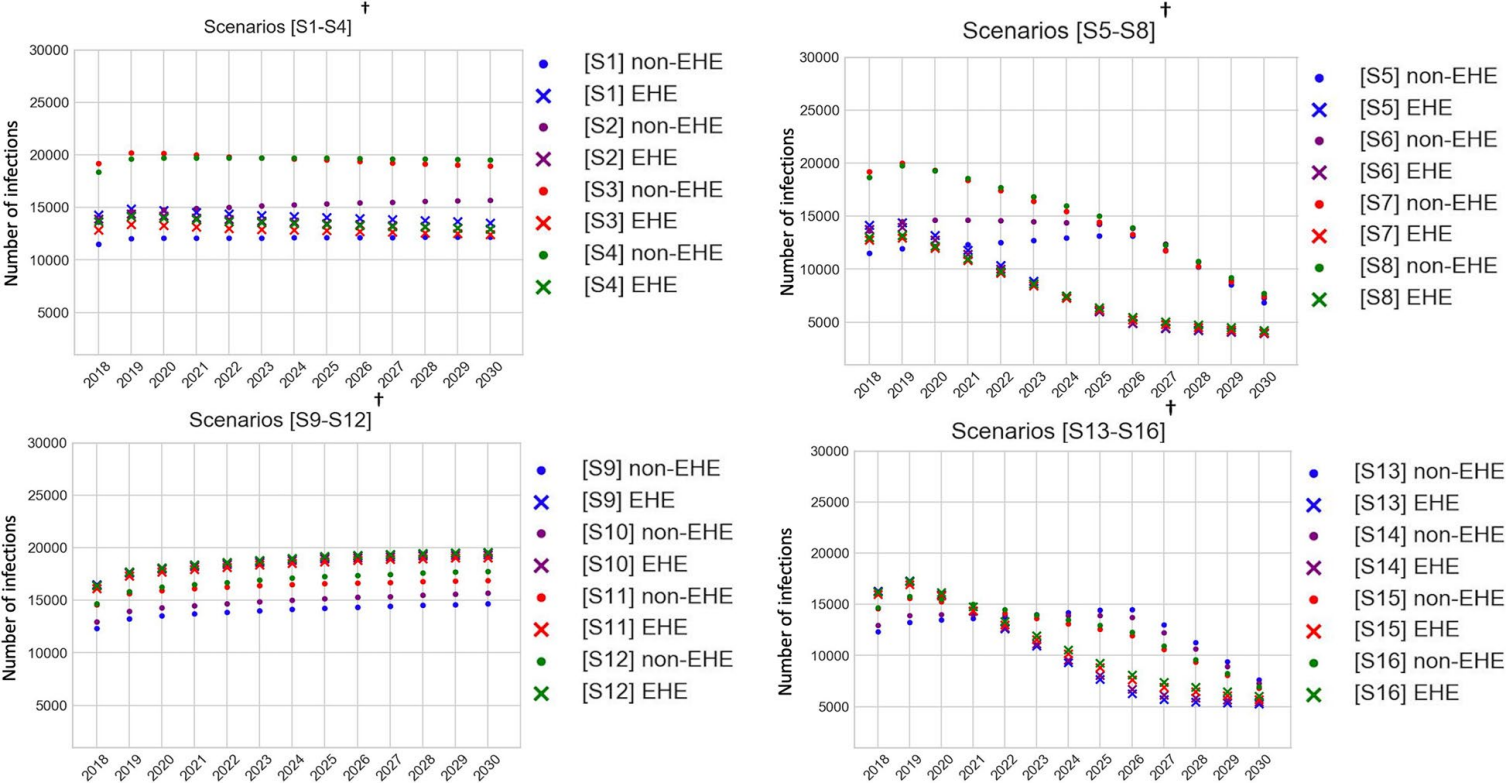
Comparing annual incidence between 16 scenarios simulated in Jurisdictional-Model, aggregated by EHE and non-EHE jurisdictions. ^†^Scenarios S1 to S8 assume jurisdictional homogeneity; Scenarios S9 to S16 assume jurisdictional heterogeneity; Scenarios S1 to S4 and S9 to S12 assume baseline intervention; Scenarios S5 to S8 and S13 to S16 scale-up interventions as per EHE-plan

The differences in aggregated national incidence estimates between no-mixing and different levels of mixing assumptions observed in year 2018 (Fig. 3, Tables 3 and 4) predominantly resulted from the non-EHE jurisdictions (see Fig. 4, summarized in Tables 3 and 4). When assuming jurisdictional homogeneity in care, compared to no-mixing S1, incidence in S2, S3, and S4 changed by 19 to 28%, 67 to 55%, and 60 to 60%, respectively, for non-EHE jurisdictions (see ‘All’ risk group “Non-EHE” in Table 3), whereas, it changed by − 3% to − 4, − 10% to − 8%, and − 5% to − 4%, respectively, for EHE jurisdictions (see ‘All’ risk group “EHE” in Table 3), the range corresponding to years 2018 to 2030. Similarly, when assuming jurisdictional heterogeneity in care, compared to no-mixing S9, incidence in S10, S11, and S12 changed by 5 to 7%, 18 to 15%, and 19 to 21%, respectively, for non-EHE jurisdictions (see ‘All’ risk group “Non-EHE” in Table 3), whereas it changed by − 1% to − 1, − 2% to − 2, and 0% to 0%, respectively, for EHE jurisdictions (see ‘All’ risk group “EHE” in Table 3).

**Fig. 5:**
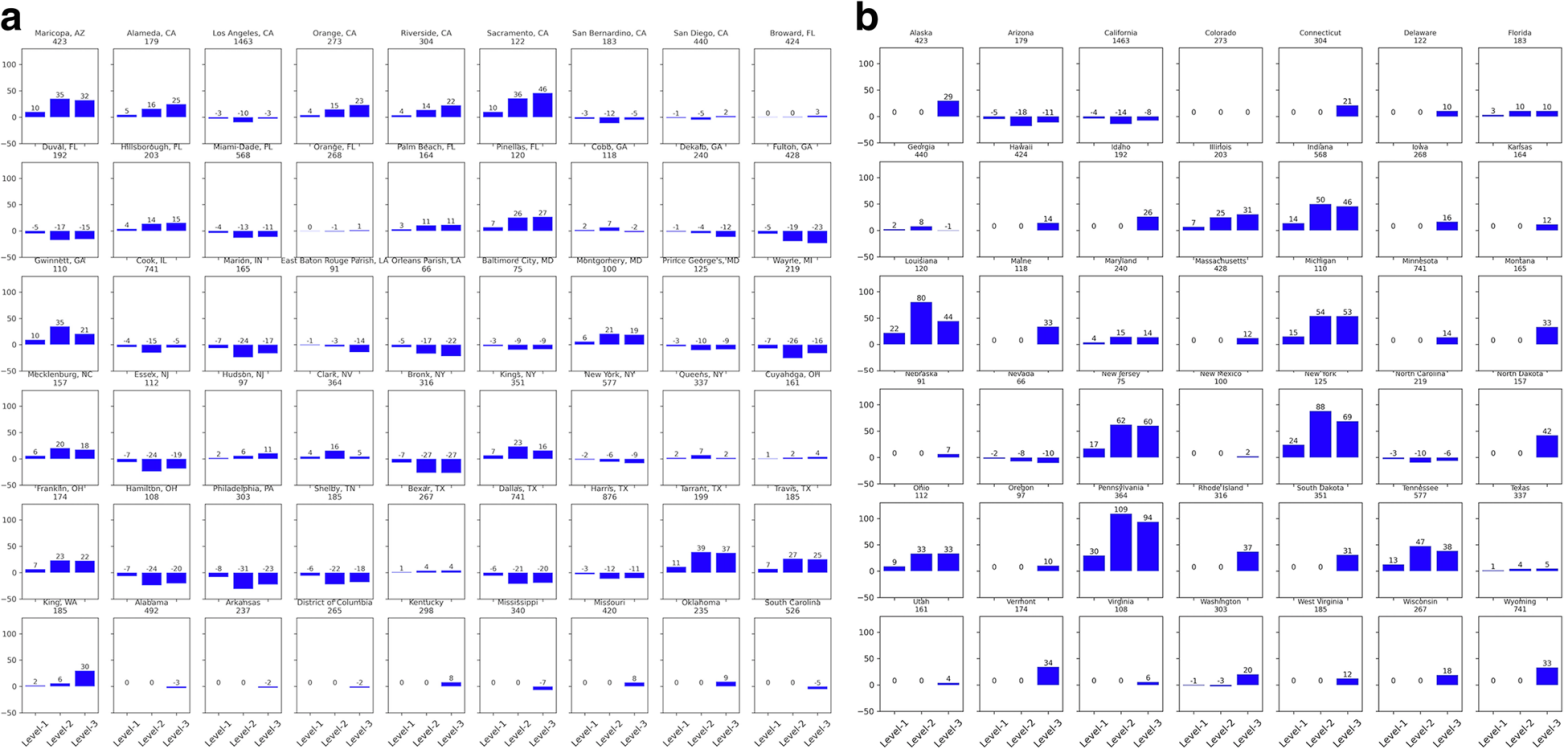
**a** Comparing percentage change in incidence estimates for no-mixing compared to mixing (EHE jurisdictions^*^, baseline, 2018). Level-1: Scenario S14; Level-2: Scenario S15; and Level-3: Scenario S16. ^*^ The title on each subplot is the EHE jurisdiction (county or state) along with values of incidence in year 2018 under the no-mixing scenario [S13]. **b** Comparing percentage change in incidence estimates for no-mixing compared to mixing (non-EHE jurisdictions^*^, baseline, 2018). Level-1: Scenario S14; Level-2: Scenario S15; and Level-3: Scenario S16. ^*^ The title on each subplot is the non-EHE jurisdiction (state) along with values of incidence in year 2018 under the no-mixing scenario [S13]

In baseline year, 2018, though overall differences in incidence between mixing assumptions were minimal when assuming heterogeneity in care, i.e., differences between scenarios S9 to S12 (Table 3) were minimal and between S13 to S16 were minimal (Table 4), the differences at the individual jurisdictions varied over a wide range. Taking differences in incidence within each jurisdiction, compared to S13, incidence in S14, S15, and S16 changed by − 8 to 30%, − 31 to 109%, and − 27 to 94%, respectively, the range corresponds to data across individual jurisdictions (see ‘All’ risk group “National” in Table 5). Further, taking only EHE jurisdictions, compared to S13, incidence in S14, S15, and S16, changed by − 8 to 11%, − 31 to 39%, and − 27 to 46%, respectively (see ‘All’ risk group “EHE” Table 5 and Fig. 5a). Considering only non-EHE jurisdictions, compared to Scenario 13, incidence in S14, S15, and S16, changed by − 5 to 30%, − 18 to 109%, and − 11 to 94%, respectively (see ‘All’ risk group “Non-EHE” in Table 5, and Fig. 5b). Differences in risk group specific incidences for S13 compared to S14, S15, and S16, for EHE and non-EHE jurisdictions had similar observations as above (Table 5, and Figs. A2a and A2b for HM, A3a and A3b for HF, and A4a and A4b for MSM in Appendix). Scenarios S9 to S12 have the same observations as above for year 2018, as they start at the same baseline values as S13 to S16, respectively.

**Table 5:** Change in jurisdiction-specific incidence^a^ (min, max^b^) in mixing scenarios compared to no-mixing (2018^e^, with jurisdictional heterogeneity^c^)

***Scenario no. ➔***		S9^**d**^ (or S13)^**e**^	S10^**d**^ (or S14)^**e**^	S11^**d**^ (or S15)^**e**^	S12^**d**^ (or S16)^**e**^
***Mixing assumption ➔***		No-mixing	Mixing level 1 (min, max) ^**b**^	Mixing level 2 (min, max) ^**b**^	Mixing level 3 (min, max) ^**b**^
***Risk group***	***Jurisdiction type***				
**All**	**National**	Ref	− 8, 30%	−31, 109%	−27, 94%
	**EHE**	Ref	− 8, 11%	− 31, 39%	− 27, 46%
	**Non-EHE**	Ref	−5, 30%	−18, 109%	− 11, 94%
**HM**	**National**	Ref	−9, 63%	− 37, 269%	− 31, 221%
	**EHE**	Ref	− 9, 13%	− 37, 56%	− 31, 43%
	**Non-EHE**	Ref	0, 63%	0, 269%	8, 221%
**HF**	**National**	Ref	− 8, 36%	− 27, 125%	− 23, 99%
	**EHE**	Ref	−8, 9%	− 27, 31%	− 23, 25%
	**Non-EHE**	Ref	−4, 36%	− 14, 125%	− 11, 99%
**MSM**	**National**	Ref	− 9, 24%	− 31, 84%	− 34, 71%
	**EHE**	Ref	−9, 12%	− 31, 44%	− 34, 52%
	**Non-EHE**	Ref	−6, 24%	−22, 84%	−17, 71%

*HM* Heterosexual males, *HF* Heterosexual females, *MSM* Men who have sex with men

National: aggregate of all EHE and non-EHE jurisdictions; EHE: aggregate of all EHE jurisdictions; Non-EHE: aggregate of all non-EHE jurisdictions;

^a^ Jurisdiction-specific % change in incidence in mixing compared to no-mixing scenario = 100 × (mixing scenario – no-mixing scenario)/mixing scenario)

^b^ Values presented are the range (minimum, maximum) across jurisdictions for year 2018

^c^ Scenarios S9 to S12 and Scenarios S13 to S16 assume jurisdictional heterogeneity

^d^ Scenarios S9 to S12 (baseline intervention) and ^e^ Scenarios S13 to S16 (EHE plan intervention) start at same baseline using 2018 care metrics (HIV-diagnosis rate, care-drop-out rate, and PrEP coverage)

A consequence of the differences in the jurisdictional-level incidence estimates is that the jurisdiction-level decisions inferred from the model would vary based on our mixing assumption. We summarize HIV-test intervals across jurisdictions into two cohorts: interval <  2 years, and interval between 2 and 4 years (Table 6). When test interval was < 2 years, compared to S13, test intervals in S14, S15, and S16, changed by − 23 to 15%, − 44 to 53%, and − 45 to 48%, respectively, the range corresponding to the minimum and maximum changes over all years, risk groups, and jurisdictions (see ‘All’ risk group “National” in Table 5). Representing these in test intervals, suppose S13 on average suggests testing every 1 year, S14, S15, and S16, would suggest testing every 0.8 to 1.2 years, 0.6 to 1.5 years, and 0.6 to 1.5 years, respectively. When test interval was 2–4 years, compared to S13, test intervals in S14, S15, and S16, changed by − 14 to 15%, − 33 to 60%, and − 28 to 37%, respectively, the range is the minimum and maximum changes over all years, risk groups, and jurisdictions (see ‘All’ risk group “National” in Table 6). Representing these in test intervals, suppose S13 on average suggests testing every 3 years, S14, S15, and S16, would suggest testing every 2.6 to 3.5 years, 2 to 4.8 years, and 2.2 to 4.1 years, respectively. These changes in testing intervals from changes in mixing assumptions were similar in both EHE and non-EHE jurisdictions.

**Table 6:** Percentage change in HIV-test intervals^a^ (min, max^b^) in mixing scenarios compared to no-mixing (EHE plan)

***Year ➔***			Mid intervention year (2022 for EHE and 2028 for non-EHE) ^**d**^				Target year (2025 for EHE and 2030 for non-EHE) ^**d**^			
***Scenario no. ➔***			S13	S14	S15	S16	S13	S14	S15	S16
***Mixing assumption ➔***			No-mixing	Mixing level 1	Mixing level 2	Mixing level 3	No-mixing	Mixing level 1	Mixing level 2	Mixing level 3
***Risk group***	***Jurisdicton type***	***Test interval***								
**MSM**	**EHE**	**< 2 years**	Ref	−14, 9%	−32, 25%	− 35, 20%	Ref	−23, 5%	−44, 13%	− 45, 12%
		**2–4 years**	Ref	−9, 10%	− 23, 36%	− 20, 34%	Ref	2, 10%	4, 34%	6, 30%
	**Non-EHE**	**< 2 years**	Ref	−2, 15%	0, 53%	−16, 48%	Ref	− 4, 13%	− 7, 50%	− 14, 37%
		**2–4 years**	Ref	10, 15%	34, 60%	36, 37%	Ref	*	*	*
**HF**	**EHE**	**< 2 years**	Ref	−12, 6%	−29, 19%	− 28, 21%	Ref	− 21, 4%	− 43, 11%	− 40, 13%
		**2–4 years**	Ref	−9, 9%	− 23, 35%	− 22, 28%	Ref	−14, 8%	− 33, 28%	− 28, 28%
	**Non-EHE**	**< 2 years**	Ref	−3, 11%	− 3, 39%	−15, 36%	Ref	− 5, 9%	− 11, 30%	− 14, 26%
		**2–4 years**	Ref	− 5, 6%	−8, 19%	− 14, 18%	Ref	−7, 4%	− 15, 12%	− 14, 12%
**HM**	**EHE**	**< 2 years**	Ref	−8, 5%	− 25, 22%	−26, 20%	Ref	− 11, 6%	− 27, 31%	− 29, 32%
		**2–4 years**	Ref	− 9, 8%	− 29, 36%	−25, 36%	Ref	− 5, 7%	− 19, 30%	− 15, 33%
	**Non-EHE**	**< 2 years**	Ref	−3, 11%	− 7, 41%	−17, 30%	Ref	−12, 10%	− 28, 32%	− 26, 23%
		**2–4 years**	Ref	− 13, 7%	− 28, 24%	− 27, 27%	Ref	*	*	*
**All**	**National**	**< 2 years**	Ref	− 14, 15%	− 32, 53%	− 45, 48%	Ref	− 23, 13%	− 44, 50%	− 45, 37%
		**2–4 years**		−13,15%	− 29, 60%	− 27, 37%		−14, 10%	− 33, 34%	−28, 37%

*HM* Heterosexual males, *HF* Heterosexual females, *MSM* Men who have sex with men

National: aggregate of all EHE and non-EHE jurisdictions; EHE: aggregate of all EHE jurisdictions; Non-EHE: aggregate of all non-EHE jurisdictions;

^a^ % change in HIV-testing interval estimate in mixing scenarios compared to no-mixing scenario and calculated as 100 × (mixing scenario – no-mixing scenario)/mixing scenario)

^b^ Values presented are the range (minimum, maximum) across the jurisdiction type for the specific time interval cohort (i.e., < 2 years (minimum value was 6 months) or 2–4 years)

^c^ Scenarios 13 to 16 scale-up care metrics (HIV-diagnosis rate, care-drop-out rate, and PrEP coverage) to reach EHE targets by 2025 for EHE jurisdictions and by 2030 for non-EHE jurisdictions

^d^ Results presented for mid-intervention years (2022 for EHE and 2028 for non-EHE) and target year (2025 for EHE and 2030 for non-EHE)

^*^ No instances/scenarios were found where interval was 2–4 years

The estimated levels of retention-in-care were similar across S13 to S16, and high (ranging from 93.5 to 100%), suggesting the need for highly effective retention-in-care programs to achieve the EHE targets.

For EHE jurisdictions, the cumulative reduction in incidence (over the period 2018 to 2030) in EHE scenarios compared to baseline scenarios were similar across jurisdictional mixing and jurisdictional heterogeneity assumptions (Table 7). However, for non-EHE jurisdictions, reduction in incidence were similar across jurisdictional heterogeneity assumptions but different across jurisdictional mixing assumptions. In non-EHE jurisdictions, while the expected incidence reduction in no-mixing assumption was 5% when assuming jurisdictional homogeneity in care (and 9% when assuming jurisdictional heterogeneity in care), the incidence reductions in level-1 mixing was 14% (and 15%), level-2 mixing was 24% (and 24%), and level-3 mixing was 23% (and 25%) (Table 7).

**Table 7:** Percentage reduction in cumulative incidence^†^ (2018–2030) (EHE plan^⁋^ compared to baseline intervention^§^)

***Scenario no. ➔ (reference scenario)***		S5^**⁋**^ (ref S1^**§**^)	S6^**⁋**^ (ref S2^**§**^)	S7^**⁋**^ (ref s3^**§**^)	S8^**⁋**^ (ref S4^**§**^)	S13^**⁋**^ (ref S9^**§**^)	S14^**⁋**^ (ref S10^**§**^)	S15^**⁋**^ (ref S11^**§**^)	S16^**⁋**^ (ref S12^**§**^)
***Care assumption ➔***		Homogeneity in care across jurisdictions*				Heterogeneity in care across jurisdictions^**^			
***Mixing assumption ➔***		No-mixing	Mixing level 1	Mixing level 2	Mixing level 3	No-mixing	Mixing level 1	Mixing level 2	Mixing level 3
***Risk group***	***Jurisdiction type***								
**All**	**National**	−24%	− 27%	− 30%	− 30%	− 30%	−31%	− 33%	−33%
	**EHE**	−41%	− 40%	−38%	− 40%	−45%	− 44%	−41%	− 41%
	**Non-EHE**	−5%	−14%	− 24%	− 23%	− 9%	− 15%	− 24%	−25%
**HM**	**National**	− 23%	− 24%	− 27%	− 27%	− 25%	− 27%	− 30%	− 30%
	**EHE**	−36%	− 35%	− 34%	− 37%	− 40%	− 39%	− 37%	− 37%
	**Non-EHE**	−4%	−12%	− 22%	− 20%	− 9%	− 15%	− 25%	− 25%
**HF**	**National**	−24%	− 25%	− 28%	− 28%	− 25%	−27%	−30%	− 30%
	**EHE**	−38%	−38%	− 36%	− 38%	− 43%	− 42%	− 40%	−39%
	**Non-EHE**	−3%	− 12%	−21%	− 20%	− 8%	− 14%	− 22%	− 23%
**MSM**	**National**	−25%	− 27%	− 31%	−31%	− 32%	− 33%	−35%	− 35%
	**EHE**	− 43%	−42%	− 40%	− 41%	−47%	− 45%	−42%	− 41%
	**Non-EHE**	−5%	− 14%	− 25%	− 24%	− 10%	−16%	− 25%	− 26%

*HM* Heterosexual males, *HF* Heterosexual females, *MSM* Men who have sex with men

National: aggregate of all EHE and non-EHE jurisdictions; EHE: aggregate of all EHE jurisdictions; Non-EHE: aggregate of all non-EHE jurisdictions;

^†^ % reduction in cumulative incidence in EHE plan scenarios^⁋^ compared to its corresponding baseline scenario^§^ and calculated as 100 × (EHE plan scenario-baseline scenario)/ baseline scenario)

^§^ Scenarios 1 to 4 and 9 to 12 keep care metrics (HIV-diagnosis rate, care-drop-out rate, and PrEP coverage) fixed at the 2018 baseline values for the full duration of the simulation

^⁋^ Scenarios 5 to 8 and 13 to 16 scale-up care metrics (HIV-diagnosis rate, care-drop-out rate, and PrEP coverage) to reach EHE targets by 2025 for EHE jurisdictions and by 2030 for non-EHE jurisdictions

^*^ Scenarios 5 to 8 and 1 to 4 assume jurisdictional homogeneity

** Scenarios 9 to 12 and 13 to 16 assume jurisdictional heterogeneity

Compared to incidence in 2019, none of the EHE-plan-intervention scenarios (S5 to S8 or S9 to S12) could reduce incidence by 75% by 2025 or 90% by 2030, as aimed for in the EHE plan. When considering jurisdictional heterogeneity (S9 to S12), aggregated incidence in EHE jurisdictions in 2018 were similar or higher than aggregated incidence in non-EHE jurisdictions. With intervening in EHE jurisdictions as per the EHE-plan, aggregated incidence in EHE jurisdictions significantly reduced over the period 2019 to 2025 (by ~ 43% in S16). Because of continuation of the baseline-intervention up to 2025 in non-EHE jurisdictions, its aggregated incidence change over the period 2019 to 2025 was minimal, its incidence surpassing that in the EHE jurisdictions by the end of 2024. Over the period 2019 to 2025, though non-EHE jurisdictions had some reductions in incidence in scenarios with mixing (~ 11% in S16) benefiting from the interventions in EHE, incidence in EHE jurisdictions increased because of the mixing, thus negating the overall benefits. As a result, by the end of 2025, the reduction in national aggregated reduction in incidence was 28% (in S16). The reduction in national aggregated incidence by 2030 compared to 2019 was about 58% (in S16). Note that the EHE plan is to first focus on only EHE jurisdictions for the first phase (2019 to 2025) and then non-EHE jurisdictions in second phase (2025 to 2030), i.e., scaling-up interventions over period 2019 to 2030 for EHE jurisdictions and period 2025 to 2030 for non-EHE jurisdictions. Instead, if we scale-up interventions over period 2019 to 2025 in all jurisdictions, both EHE and non-EHE, and keep it constant thereafter up to 2030, we would achieve a reduction in national incidence of about 52% by 2025 and 67% by 2030 (see Fig. A5 in Appendix).

**Fig. 6:**
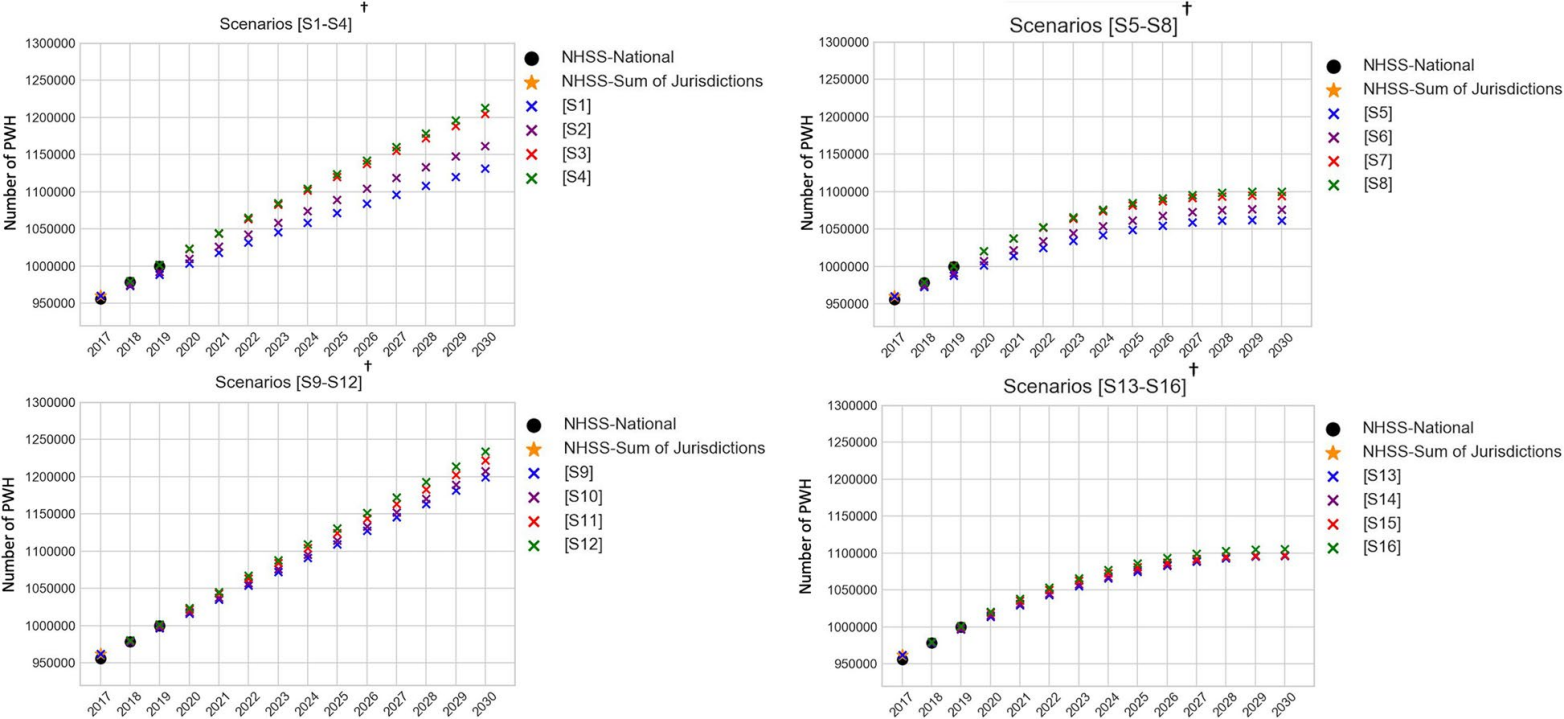
Comparing PWH projections between 16 scenarios simulated in Jurisdictional-Model and NHSS estimates^*^. PWH: people with HIV; NHSS: National HIV Surveillance System; NHSS-National: national level estimates from NHSS; NHSS-Sum of Jurisdictions: sum of jurisdiction level estimates from NHSS. ^*^Using period 2018 to 2019 to verify that Jurisdictional-Model simulated estimates generate PWH in magnitudes similar to NHSS estimates; and using period 2020 to 2030 to observe differences between Jurisdictional-Model scenarios S1 to S16. NHSS-Sum of jurisdictions value (denoted by a shaded circle in the figure) and NHSS-National value (denoted by a star in the figure) for year 2018 is compared with model generated estimate. ^†^ Scenarios S1 to S8 assume jurisdictional homogeneity; Scenarios S9 to S16 assume jurisdictional heterogeneity; Scenarios S1 to S4 and S9 to S12 assume baseline intervention; Scenarios S5 to S8 and S13 to S16 scale-up interventions as per EHE-plan

The corresponding changes in prevalence (number of people with HIV) estimates over the period 2017 to 2030 are presented in Fig. 6 along with the national surveillance estimates (‘NHSS-National’) for years 2017–2019 and sum of the 96 jurisdictions for 2017 (‘NHSS-Sum of jurisdictions’). Simulated estimates of prevalence match close to the surveillance estimates for years 2017 to 2019, however, following from the changes in incidence over time (Fig. 2), prevalence were most sensitive to jurisdictional mixing in Scenarios S1 to S4 which assumed homogeneity in care and baseline interventions.

## Discussions

In this study, we developed a compartmental model to project the national HIV epidemic in the U.S. We first developed a National-Model, which calibrated risk group and age group specific sexual behavioral parameters at the national level for years 2011–2018. The National-model was not split into individual jurisdictions. We then developed a Jurisdictional-Model, which split the population into 96 jurisdictions, and simulated the national HIV epidemic in the U.S. from 2018 to 2030 as a composition of 96 local epidemics. We used the Jurisdictional-Model to evaluate the sensitivity of jurisdictional mixing and jurisdictional heterogeneity in care on aggregated-national and jurisdiction-specific HIV incidence estimates, and the corresponding intervention decisions, such as HIV-testing interval and retention-in-care, inferred from the model.

We believe this is the first model that simulates the U.S. national HIV epidemic through simulating interacting individual sub-geographical jurisdictions. Previous models in the literature have either focused on modeling jurisdictions individually and independently of other jurisdictions or aggregated into a national model assuming homogeneity among the national population. Though the scope of the models in the literature were different than that presented here, there were some common observations in terms of intervention findings and more broadly model development from our study that matched with that in the literature. Specifically, that the EHE targets cannot be achieved by continuation of status-quo interventions [9, 10, 13], the heterogeneity in epidemics and care uptakes across jurisdictions generate a need for more tailored interventions that may vary across jurisdictions [8–10, 13], the lack of data for developing comprehensive models for every jurisdiction, and the role that models could play in improving data collection by identifying most sensitive parameters to inform data priorities [8, 10].

In addition to observations on heterogeneity across populations, that generated some of the work in the literature, our work was additionally motivated by the observations in partnership mixing between jurisdictions, in both sexual behavior surveys and phylogenetic analyses of clusters [19–24]. However, there are challenges to developing a model that is fully representative of all jurisdictions. First, large-scale national level data for partnership mixing such as from behavioral surveys are limited to small populations. Second, they are infeasible to generate for every jurisdiction pair. Third, though current model types do not simulate jurisdictional interactions, it is not clear if jurisdictional interactions are significant enough to alter decisions, and thus the need for more comprehensive jurisdictional models over current model types.

Therefore, we focused the scope of this work on conducting extensive sensitivity analyses to evaluate the following: 1) the sensitivity of jurisdictional mixing on the national HIV incidence estimates under baseline intervention, i.e., if care and PrEP coverage continued at 2018 levels, would incidence estimates from the model change based on jurisdictional mixing assumptions, and would they be different when assuming jurisdictional homogeneity versus jurisdictional heterogeneity; 2) the sensitivity of jurisdictional mixing on the projected national HIV incidence estimates and incidence reductions when simulating the EHE plan interventions, i.e., if the EHE care-continuum and PrEP targets were reached, would the corresponding incidence estimates and reductions compared to baseline-intervention vary based on mixing assumptions, and would they be different when assuming jurisdictional homogeneity versus jurisdictional heterogeneity; and 3) the sensitivity of jurisdictional mixing on the intervention decisions inferred through simulated estimates under the EHE plan, i.e., would model outcomes for HIV-testing frequency and retention-in-care to achieve the EHE plan’s targets vary based on jurisdictional mixing assumptions. We discuss our observations for each below.

Under baseline-intervention, our results suggest that nationally aggregated incidence (and thus PWH estimates) were sensitive to both jurisdictional mixing and jurisdictional heterogeneity assumptions, i.e., keeping one fixed and varying the other resulted in different incidence estimates. Comparing across mixing assumptions, nationally aggregated incidence were more sensitive to jurisdictional mixing when assuming jurisdictional homogeneity in care than when assuming jurisdictional heterogeneity in care. Incidence aggregated over non-EHE jurisdictions were more sensitive to jurisdictional mixing assumptions than incidence aggregated over EHE jurisdictions. However, within each jurisdiction, incidence estimates in both EHE and non-EHE jurisdictions were sensitive to jurisdictional mixing assumptions irrespective of jurisdictional homogeneity or heterogeneity in care.

Under EHE intervention, though nationally aggregated incidence estimates were sensitive to jurisdictional mixing and heterogeneity in 2018 (baseline intervention starts in 2018), as incidence decreased over time with implementation of the EHE plan, the sensitivity decreased. In 2030, incidence estimates were similar across all scenarios. However, the cumulative incidence reduction in 2030 compared to 2018 were sensitivity, with variations by EHE and non-EHE similar to that in baseline-intervention (discussed above). Cumulative incidence reductions in EHE plan compared to baseline-intervention, calculated within each mixing and care assumption, had similar estimates except for aggregated non-EHE jurisdictions, which were sensitivity to jurisdictional mixing.

Under EHE intervention, jurisdictional-level decisions related to HIV-testing intervals, inferred from the model to reach the EHE targets, were sensitive to jurisdictional mixing and jurisdictional heterogeneity assumptions. Comparing across jurisdictional mixing assumptions, compared to the no-mixing scenario, while there were differences in HIV-test intervals even at the lowest mixing-level, the differences were more predominant in the higher-mixing scenarios. These results suggest that when modeling jurisdictions independently, understanding the magnitude and accounting for the mixing outside jurisdictions could lead to better decisions.

From the above three observations, we can infer the following. If a model’s goal is to estimate changes in incidence in EHE plan compared to baseline intervention, and the focus is on aggregated national level incidence estimates, jurisdictional mixing and heterogeneity assumptions play a minor role. Though the aggregated national incidence estimates were not always sensitive to jurisdictional mixing,within each jurisdiction, incidence estimates, and corresponding model-inferred decisions were sensitive, for both EHE and non-EHE jurisdictions. Therefore, if a model’s goal is to infer jurisdiction-specific decisions, then, in developing jurisdiction-specific models, in addition to using jurisdiction specific care and demographics, accounting for outside mixing can help improve model-based analyses.

Our results also suggest that increased testing, care-retention, and PrEP as per the EHE plan may not achieve the 90% incidence reduction goal of the EHE plan by 2030. Diagnose (through increased testing), treat (through care retention), and prevent (through PrEP), the interventions modeled in our analyses, are three of the four strategic pillars of the EHE plan. The fourth pillar is respond, through phylogenetic network cluster-based detection and response of new outbreaks. Our results suggest that use of the fourth pillar would be key to achieving the overall incidence reduction goals of the EHE.

Our model has limitations. Our model only simulates sexual partnership and does not model transmission due to injecting drug use. Jurisdiction-specific data on care or mixing are not fully available, and thus, our model is currently limited to be used as a tool to evaluate its sensitivity to model-inferred decisions. Sexual partnership mixing within and outside jurisdictions are likely influenced by several factors including individual preferences and social conditions [19–23], and thus vary by jurisdiction, which we did not model. We assumed there are no change in behavioral, demographic, and disease transmission factors over time. Several simplifying assumptions were made in model development for partnership mixing (which remained consistent across geographies) as jurisdiction-specific partnership mixing and sexual behavior data by age and risk group were not available, which could also impact the outcomes. However, as the scope of the analyses was to evaluate the sensitivity of jurisdictional mixing and heterogeneity, and thus the need to inform data collection and model development, and not to infer decisions, we believe, the above assumptions are reasonable. Our model is currently limited to be used as a tool to evaluate its sensitivity to model-inferred decisions. However, if data were to become available, our model can also serve as a decision analytic tool.

## Conclusions

We developed a Jurisdictional-Model, which split the population into 96 jurisdictions, and simulated the national HIV epidemic in the U.S. from 2018 to 2030 as a composition of 96 local epidemics. Such a model will help evaluate the national epidemic as a whole, while considering geographical heterogeneity in population demographics, HIV epidemic, and intervention decisions. It would be instrumental in identifying what jurisdiction-specific strategies to adopt, such as how often to test, what should be the aim for retention-in-care, and what should be the target for PrEP coverage to achieve the intended goals of the EHE plan.

However, there are data gaps to be addressed before the model can serve as a decision analytic tool. Here we focused the analyses on evaluating the sensitivity of those data gaps, specifically jurisdictional mixing and jurisdiction heterogeneity, on key outcomes of interest, with the objective to inform data collection and future model development. From observed results, we can infer that jurisdictional-level decisions inferred from the model were sensitive to jurisdictional mixing and jurisdictional heterogeneity assumptions, however, the differences were minimal under low mixing. Therefore, when modeling jurisdictions independently, understanding the magnitude and accounting for the mixing outside jurisdictions could add robustness to model inferred analyses. We can also infer that jurisdictional mixing and heterogeneity, though sensitive to jurisdiction-specific intervention decisions, were not sensitive to national aggregated estimates for incidence reductions in EHE plan compared to baseline-intervention. Our study also suggests that only increased testing, care-retention, and PrEP may not achieve 90% incidence reduction by 2030, which is the goal of the EHE. Thus, additional interventions would be necessary. Our work also highlights gaps in data on epidemic and care metrics specific to jurisdictions. Upon availability of such data, the model can serve as a decision-analytic tool to infer jurisdiction-specific intervention strategies for reaching the EHE goals nationally.

## Supplementary Information

**Additional file 1.**

## Data Availability

The datasets used in the development of the simulation are presented in the Appendix and data generated and/or analysed during the current study are available from the corresponding author on reasonable request.

## Abbreviations

EHE: Ending the HIV epidemic
HIV: Human immunodeficiency virus
U.S.: United States
PWH: People with HIV
ART: Antiretroviral therapy treatment
PrEP: Pre-exposure prophylaxis
VLS: Viral load suppression
MSM: Men who have sex with men
HM: Heterosexual males
HF: Heterosexual females
NHSS: National HIV Surveillance Systems
DNA: Deoxyribonucleic acid
CD4: Cluster of differentiation 4

## References

[1] Clinical Info HIV.gov, “U.S. Statistics,” Jun. 02, 2021. https://www.hiv.gov/hiv-basics/overview/data-and-trends/statistics (Accessed 12 Aug 2021).

[2] National Institutes of Health (NIH), “HIV Treatment.” https://hivinfo.nih.gov/understanding-hiv/fact-sheets/hiv-treatment-basics (Accessed 12 Aug 2021).

[3] Centers for Disease Control and Prevention (CDC), “PrEP Effectiveness,” May 13, 2021. https://www.cdc.gov/hiv/basics/prep/prep-effectiveness.html (Accessed 20 Aug 2021).

[4] America’s HIV Epidemic Analysis Dashboard (AHEAD), “What is AHEAD?” https://ahead.hiv.gov/about (Accessed 12 Aug 2021).

[5] HIV.gov, “What Is Ending the HIV Epidemic in the U.S.?,” Jun. 02, 2021. https://www.hiv.gov/federal-response/ending-the-hiv-epidemic/overview (Accessed 12 Aug 2021).

[6] Centers for Disease Control and Prevention (CDC), “Ending the HIV Epidemic in the U.S. (EHE),” Sep. 07, 2021. https://www.cdc.gov/endhiv/jurisdictions.html?CDC_AA_refVal=https%3A%2F%2Fwww.cdc.gov%2Fendhiv%2Fpriorities.html (Accessed 14 Dec 2021).

[7] Clinical Info HIV.gov, “Prior National HIV/AIDS Strategies (2010–2021),” Dec. 01, 2021. https://www.hiv.gov/federal-response/national-hiv-aids-strategy/national-hiv-aids-strategies-2010-2021 (Accessed 17 Dec 2021).

[8] Krebs E (2019). Developing a dynamic HIV transmission model for 6 U.S. cities: an evidence synthesis. PLoS One.

[9] Nosyk B (2019). Ending the epidemic in America will not happen if the status quo continues: modeled projections for human immunodeficiency virus incidence in 6 US cities. Clin Infect Dis.

[10] Zang X (2020). Development and calibration of a dynamic HIV transmission model for 6 US cities. Med Decis Mak.

[11] Nosyk B (2020). Ending the HIV epidemic in the USA: an economic modelling study in six cities. The Lancet HIV.

[12] Krebs E (2020). Ending the HIV epidemic among persons who inject drugs: a cost-effectiveness analysis in six US cities. J Infect Dis.

[13] Zang X (2020). Can the ‘ending the HIV epidemic’ initiative transition the USA towards HIV/AIDS epidemic control?. AIDS.

[14] Nosyk B (2020). ‘Ending the epidemic’ will not happen without addressing racial/ethnic disparities in the United States human immunodeficiency virus epidemic. Clin Infect Dis.

[15] Quan AML, et al. Improving health equity and ending the HIV epidemic in the USA: a distributional cost-effectiveness analysis in six cities. The Lancet HIV. 2021:S2352301821001478. 10.1016/S2352-3018(21)00147-8. 10.1016/S2352-3018(21)00147-8 PMC8423356 34370977

[16] Fojo AT, Schnure M, Kasaie P, Dowdy DW, Shah M (2021). What will it take to end HIV in the United States?: a comprehensive, local-level modeling study. Ann Intern Med.

[17] Khatami SN, Gopalappa C, Mechanical and Industrial Engineering Department, University of Massachusetts Amherst, Amherst, MA 01003, USA (2021). A reinforcement learning model to inform optimal decision paths for HIV elimination. MBE.

[18] Khurana N (2018). Impact of improved HIV care and treatment on PrEP effectiveness in the United States, 2016–2020. JAIDS Journal of Acquired Immune Deficiency Syndromes.

[19] Gindi RM, Sifakis F, Sherman SG, Towe VL, Flynn C, Zenilman JM (2011). The geography of heterosexual partnerships in Baltimore City adults. Sex Transm Dis.

[20] Oster AM (2011). Demographic but not geographic insularity in HIV transmission among young black MSM. AIDS.

[21] Lubelchek RJ, Hoehnen SC, Hotton AL, Kincaid SL, Barker DE, French AL (2015). Transmission clustering among newly diagnosed HIV patients in Chicago, 2008 to 2011: using Phylogenetics to expand knowledge of regional HIV transmission patterns. JAIDS Journal of Acquired Immune Deficiency Syndromes.

[22] Li X (2017). Multiple introductions and onward transmission of HIV-1 subtype B strains in Shanghai, China. J Inf Secur.

[23] Gesink D (2018). Conceptualizing Geosexual archetypes: mapping the sexual travels and egocentric sexual networks of gay and bisexual men in Toronto, Canada. Sexual Trans Dis.

[24] Board AR (2020). Geographic distribution of HIV transmission networks in the United States. JAIDS Journal of Acquired Immune Deficiency Syndromes.

[25] Grey JA (2016). Estimating the population sizes of men who have sex with men in US states and counties using data from the American community survey. JMIR Public Health Surveill.

[26] “NCHHSTP AtlasPlus.” https://www.cdc.gov/nchhstp/atlas/index.htm (Accessed 04 Nov 2021).

[27] “PrEP for HIV Prevention in the U.S.” https://www.cdc.gov/nchhstp/newsroom/fact-sheets/hiv/PrEP-for-hiv-prevention-in-the-US-factsheet.html (Accessed 30 Jun 2022).

[28] Baugher AR (2021). Racial, ethnic, and gender disparities in awareness of Preexposure prophylaxis among HIV-negative heterosexually active adults at increased risk for HIV infection - 23 urban areas, United States, 2019. MMWR Morb Mortal Wkly Rep.

[29] United States Census Bureau (2020). Annual County resident population estimates by age, sex, race, and Hispanic origin: April 1, 2010 to July 1, 2019.

[30] America’s HIV Epidemic Analysis Dashboard (AHEAD), “FAQs,” EHE Goals: What are the 2025 goals and 2030 goals for each of the indicators? https://ahead.hiv.gov/faqs (Accessed 20 Aug 2021).

[31] Clinical Info HIV.gov, “Recommendations for the Use of Antiretroviral Drugs in Pregnant Women with HIV Infection and Interventions to Reduce Perinatal HIV Transmission in the United States,” Dec. 29, 2020. https://clinicalinfo.hiv.gov/en/guidelines/perinatal/prep (Accessed 8 Nov 2021).

