# Supplementary material for: Evaluating the sensitivity of jurisdictional heterogeneity and jurisdictional mixing in national level HIV prevention analyses: context of the U.S. ending the HIV epidemic plan
Source: BMC Med Res Methodol. 2022 Nov 26;22:304. doi: 10.1186/s12874-022-01756-w (PMC9701422; doi:10.1186/s12874-022-01756-w)
Supplement: Supplementary file 1 — Additional file 1. [file 12874_2022_1756_MOESM1_ESM.pdf]

Appendix: Evaluating the sensitivity of jurisdictional heterogeneity and jurisdictional mixing in national-level HIV prevention analyses: Context of the U.S. Ending the HIV Epidemic plan.

Hanisha Tatapudi, Ph.D.[1], and Chaitra Gopalappa, Ph.D.[2]

[1] Department of Industrial and Management System Engineering, University of South Florida, Tampa, Florida, USA.

[2] Mechanical and Industrial Engineering, University of Massachusetts Amherst, Amherst, Massachusetts, USA.

Corresponding author: Chaitra Gopalappa

Email: chaitrag@umass.edu

Table of Contents

A1: Jurisdictions modeled ***3***

A2: Disease, care continuum, and death related parameters for both compartmental models ***6***

A3: Estimation of incidence using Bernoulli Equation ***7***

National-Model **7**

Jurisdictional-Model **9**

A4: Estimation of diagnosis and retention-in-care rates ***12***

A4.1 Estimation of diagnosis rates **13**

A4.2 Estimation of dropout rates **14**

A5: Estimation of jurisdiction-specific proportion aware categorized by risk group ***15***

A1: Jurisdictions modeled

Table A1: List of EHE* and non-EHE jurisdictions (counties and states) modeled [1]

List of counties from CDC Atlas					***List of States and dependent areas from CDC Atlas***			
S. no.	*State*	*County*	***FIPS***	*Modeled/ why not modeled*	*S.no.*	*State*	***FIPS***	*Modeled/ why not modeled*
1.	CA	Alameda County	**6001**	Yes	1.	Alabama	**1**	Yes
2.	MD	Baltimore City	**24510**	Yes	2.	Alaska	**2**	Yes
3.	TX	Bexar County	**48029**	Yes	3.	Arizona	**4**	Yes
4.	NY	Bronx County	**36005**	Yes	4.	Arkansas	**5**	Yes
5.	FL	Broward County	**12011**	Yes	5.	California	**6**	Yes
6.	NV	Clark County	**32003**	Yes	6.	Colorado	**8**	Yes
7.	GA	Cobb County	**13067**	Yes	7.	Connecticut	**9**	Yes
8.	IL	Cook County	**17031**	Yes	8.	Delaware	**10**	Yes
9.	OH	Cuyahoga County	**39035**	Yes	9.	District of Columbia	**11**	Yes
10.	TX	Dallas County	**48113**	Yes	10.	Florida	**12**	Yes
11.	GA	Dekalb County	**13089**	Yes	11.	Georgia	**13**	Yes
12.	DC	District of Columbia	**11001**	Modeled as a state	12.	Hawaii	**15**	Yes
13.	FL	Duval County	**12031**	Yes	13.	Idaho	**16**	Yes
14.	LA	East Baton Rouge Parish	**22033**	Yes	14.	Illinois	**17**	Yes
15.	NJ	Essex County	**34013**	Yes	15.	Indiana	**18**	Yes
16.	OH	Franklin County	**39049**	Yes	16.	Iowa	**19**	Yes
17.	GA	Fulton County	**13121**	Yes	17.	Kansas	**20**	Yes
18.	GA	Gwinnett County	**13135**	Yes	18.	Kentucky	**21**	Yes
19.	OH	Hamilton County	**39061**	Yes	19.	Louisiana	**22**	Yes
20.	TX	Harris County	**48201**	Yes	20.	Maine	**23**	Yes
21.	FL	Hillsborough County	**12057**	Yes	21.	Maryland	**24**	Yes
22.	NJ	Hudson County	**34017**	Yes	22.	Massachusetts	**25**	Yes

23.	WA	King County	**53033**	Yes	23.	Michigan	**26**	Yes
24.	NY	Kings County	**36047**	Yes	24.	Minnesota	**27**	Yes
25.	CA	Los Angeles County	**6037**	Yes	25.	Mississippi	**28**	Yes
26.	AZ	Maricopa County	**4013**	Yes	26.	Missouri	**29**	Yes
27.	IN	Marion County	**18097**	Yes	27.	Montana	**30**	Yes
28.	NC	Mecklenburg County	**37119**	Yes	28.	Nebraska	**31**	Yes
29.	FL	Miami-Dade County	**12086**	Yes	29.	Nevada	**32**	Yes
30.	MD	Montgomery County	**24031**	Yes	30.	New Hampshire	**33**	Data suppressed
31.	NY	New York County	**36061**	Yes	31.	New Jersey	**34**	Yes
32.	FL	Orange County	**12095**	Yes	32.	New Mexico	**35**	Yes
33.	CA	Orange County	**6059**	Yes	33.	New York	**36**	Yes
34.	LA	Orleans Parish	**22071**	Yes	34.	North Carolina	**37**	Yes
35.	FL	Palm Beach County	**12099**	Yes	35.	North Dakota	**38**	Yes
36.	PA	Philadelphia County	**42101**	Yes	36.	Ohio	**39**	Yes
37.	FL	Pinellas County	**12103**	Yes	37.	Oklahoma	**40**	Yes
38.	MD	Prince George's County	**24033**	Yes	38.	Oregon	**41**	Yes
39.	NY	Queens County	**36081**	Yes	39.	Pennsylvania	**42**	Yes
40.	CA	Riverside County	**6065**	Yes	40.	Rhode Island	**44**	Yes
41.	CA	Sacramento County	**6067**	Yes	41.	South Carolina	**45**	Yes
42.	CA	San Bernardino County	**6071**	Yes	42.	South Dakota	**46**	Yes
43.	CA	San Diego County	**6073**	Yes	43.	Tennessee	**47**	Yes
44.	CA	San Francisco County	**6075**	Data suppressed	44.	Texas	**48**	Yes
45.	PR	San Juan Municipio	**72127**	Population demographic data not available for counties and state	45.	Utah	**49**	Yes
46.	TN	Shelby County	**47157**	Yes	46.	Vermont	**50**	Yes

47.	MA	Suffolk County	**25025**	Data suppressed	47.	Virginia	**51**	Yes
48.	TX	Tarrant County	**48439**	Yes	48.	Washington	**53**	Yes
49.	TX	Travis County	**48453**	Yes	49.	West Virginia	**54**	Yes
50.	MI	Wayne County	**26163**	Yes	50.	Wisconsin	**55**	Yes
					51.	Wyoming	**56**	Yes
					52.	American Samoa	**60**	Data not available
					53.	Guam	**66**	Data not available
					54.	Northern Mariana Islands	**69**	Data not available
					55.	Puerto Rico	**72**	Demographic data not available
					56.	U.S. Virgin Islands	**78**	Data not available

CDC: Centers for Disease Control and Prevention
* EHE jurisdictions are in blue; states that have EHE counties within them are excluded

A2: Disease, care continuum, and death related parameters for both compartmental models

Table A2: Rates of care continuum and disease progression used in the matrix G_t

From§	*To*§	*Progression type*	*Rate**	*Source*
(A-U) (1)	(A-ANA) (2)	Care	Diagnosis rate† × $\theta_{d,risk}$	Estimated
	(U) >500 (3)	Disease	5.88	[3][4]
(A-ANA) (2)	(ANA) >500 (4)	Disease	5.88	[3][4]
(U) >500 (3)	(ANA) >500 (4)	Care	Diagnosis rate† × (1-linkage to care‡) × $\theta_{d,risk}$	Estimated
	(ANV) >500 (5)	Care	Diagnosis rate† × linkage to care‡ × $\theta_{d,risk}$	Estimated
	(U) 351-500 (7)	Disease	0.286	[3,5–8]
(ANA) >500 (4)	(ANV) >500 (5)	Care	0.5	[9]
	(ANA) 351-500 (8)	Disease	0.286	[3,5–8]
(ANV) >500 (5)	(ANA) >500 (4)	Care	Dropout rate† × φ_d	Estimated
	(VLS) >500 (6)	Care	1.33	[10]
	(ANV) 351-500 (9)	Disease	0.026	[3]
(VLS) >500 (6)	(ANA) >500 (4)	Care	Dropout rate† × φ_d	Estimated
(U) 351-500 (7)	(ANA) 351-500 (8)	Care	Diagnosis rate† × (1-linkage to care‡) × $\theta_{d,risk}$	Estimated
	(ANV) 351-500 (9)	Care	Diagnosis rate† × linkage to care‡ × $\theta_{d,risk}$	Estimated
	(U) 201-350 (11)	Disease	0.286	[3,5–8]
(ANA) 351-500 (8)	(ANV) 351-500 (9)	Care	0.5	[9]
	(ANA) 201-350 (12)	Disease	0.286	[3,5–8]
(ANV) 351-500 (9)	(ANA) 351-500 (8)	Care	Dropout rate† × φ_d	Estimated
	(VLS) 351-500 (10)	Care	1.33	[10]
	(ANV) 201-350 (13)	Disease	0.026	[3]
(VLS) 351-500 (10)	(VLS) >500 (6)	Disease	0.385	[3]
	(ANA) 351-500 (8)	Care	Dropout rate† × φ_d	Estimated
(U) 201-350 (11)	(ANA) 201-350 (12)	Care	Diagnosis rate† × (1-linkage to care‡) × $\theta_{d,risk}$	Estimated
	(ANV) 201-350 (13)	Care	Diagnosis rate† × linkage to care‡ × $\theta_{d,risk}$	Estimated
	(U) <200 (15)	Disease	0.33	[3,6,11–14]
(ANA) 201-350 (12)	(ANV) 201-350 (13)	Care	0.5	[9]
	(ANA) <200 (16)	Disease	0.33	[3,6,11–14]
(ANV) 201-350 (13)	(ANA) 201-350 (12)	Care	Dropout rate† × φ_d	Estimated
	(VLS) 201-350 (14)	Care	1.33	[10]
	(ANV) <200 (17)	Disease	0.026	[3]
(VLS) 201-350 (14)	(VLS) 351-500 (10)	Disease	0.385	[3]
	(ANA) 201-350 (12)	Care	Dropout rate† × φ_d	Estimated
(U) <200 (15)	(ANA) <200 (16)	Care	Diagnosis rate† × (1-linkage to care‡) × $\theta_{d,risk}$	Estimated
	(ANV) <200 (17)	Care	Diagnosis rate† × linkage to care‡ × $\theta_{d,risk}$	Estimated
(ANA) <200 (16)	(ANV) <200 (17)	Care	1	[15]
(ANV) <200 (17)	(ANA) <200 (16)	Care	Dropout rate† × φ_d	Estimated
	(VLS) <200 (18)	Care	1.33	[10]
(VLS) <200 (18)	(VLS) 201-350 (14)	Disease	0.355	[3]
	(ANA) <200 (16)	Care	Dropout rate† × φ_d	Estimated

$\theta_{d,risk}$= scaling factor for diagnosis rate in disease-stage d, varies by risk group. The total scaling factor = average annual rate of diagnosis in each disease stage × (scaling factor for conventional test + scaling factor for rapid test). Scaling factor for each test type (conventional or rapid) is calculated as percentage of test type × test sensitivity × probability of notification. Data for testing types, sensitivity, notification probability, and annual diagnosis rates are obtained from [3].
φ_d= scaling factor for drop-out rate in disease-stage d. Scaling factor is 1 for all CD4 counts above 200 and 0 for CD4 below 200.
[*] Rates in table represent annual rates input to the simulation model.
[†] Diagnosis rate and Dropout rate are rates of care metrics estimated monthly for each risk group.
[‡] Data on linkage to care changes across risk groups, time, and jurisdictions [16].
[§] Numbers within parenthesis "()" refer to compartment numbers as seen in Figure 1.

Table A3: Death rates for HIV infected without ART [17]

Disease stage	***Death rate***
**CD4 <200**	0.117
**CD4 200-350**	0.024
**CD4 350-500**	0.012
**CD4 >500**	0.008
**Acute**	0.008

Table A4: Death rates for HIV infected after ART initiation by disease stages [18]

Age group	***Disease stage CD4 > 350***	***Disease stage CD4 > 200-350***	***Disease stage CD4 < 200***
**13-29**	0.004	0.005	0.015
**30-39**	0.005	0.006	0.019
**40-49**	0.006	0.008	0.025
**50-100**	0.046	0.016	0.011

A3: Estimation of incidence using Bernoulli Equation

We estimate the number of persons transitioning from the susceptible to infected compartments, i.e., the number of newly persons using a Bernoulli model, developed for both, the National-Model and the Jurisdictional-Model.

National-Model

We apply the following Bernoulli equation for calculating the number of new infections as follows.
Let,

$p_{v,x1}$ = probability of transmission for vaginal acts for risk group x_1 per sexual act,
$p_{a,x1}$ = probability of transmission for anal acts for risk group x_1 per sexual act,
ϵ = probability of condom effectiveness,
$n_{v,x1,y1}$ = number of annual vaginal acts for risk group x_1, and age group y_1 (number of acts × proportion of anal acts),
$n_{a,x1,y1}$ = number of annual anal acts for risk group x_1, and age group y_1 (number of acts × (1-proportion of anal acts)),
c_i = proportion reduction in number of unprotected acts when aware in infected compartment i,
$d_{x1,y1}$ = number of partners for risk group x_1, and age group y_1 (calculated as weighted average of median number of partners for each partnership type and proportion of partnership type),
p_c = proportion of persons having only casual partners,
p_{c+m} = proportion of persons having casual and main partners,
p_m = proportion of persons having only main partners,

c_c = proportion of condom use among casual partners,
c_m = proportion of condom use among main partners,
m_{c+m} = number of annual casual partners among persons with casual and main partnerships,
S_c = number of annual sexual acts with each casual contact (assumed 2, median between 1 and 3), and
S_{acts} = number of annual sexual acts per person.

We calculate the number of new infections in risk group x_1 and age group y_1 as

$$= S_{x1,y1}[1 - \prod_{i=1}^{18}\{\mathrm{M}_{x_1,y_1,i}\}^{q_{x1,y1,i}}] \tag{2}$$

where,
$S_{x1,y1}$ is the number of susceptible individuals in risk group x_1, and age group y_1;
$1 - \mathrm{M}_{x_1,y_1,i}$ is the transmission probability per partnership for a susceptible person in risk group x_1 and age group y_1 from interactions with an infected person in compartment i , and is calculated as ,

$$M_{x_1,y_1,i} = \left\{1 - \left[1 - \left\{\left(1 - [\bar{p}_{v,x_1}p_i]\right)^{m_{v,c}}\left(1 - [p_{v,x_1}p_i]\right)^{m_{v,c'}}\left(1 - [\bar{p}_{a,x_1}p_i]\right)^{m_{a,c}}\left(1 - [p_{a,x_1}p_i]\right)^{m_{a,c'}}\right\}\right]\right\} \tag{3}$$

$\bar{p}_{v,x1} = p_{v,x1}(1-\epsilon)$ = probability of transmission per protected sexual act (vaginal) for risk group x_1,
$\bar{p}_{a,x1} = p_{a,x1}(1-\epsilon)$ = probability of transmission per protected sexual act (anal) for risk group x_1,
p_i = factor for transmission probability based on infected compartment i,
$m_{v,c} = \frac{n_{v,x1,y1}((1-c_{x1,y1})c_i + c_{x1,y1})}{d_{x1,y1}}$ = number of annual protected sexual acts (vaginal) per partner,
$m_{v,c\prime} = \frac{n_{v,x1,y1}(1-c_{x1,y1})(1-c_i)}{d_{x1,y1}}$ = number of annual unprotected sexual acts (vaginal) per partner,
$m_{a,c} = \frac{n_{a,x1,y1}((1-c_{x1,y1})c_i + c_{x1,y1})}{d_{x1,y1}}$ = number of annual protected sexual acts (anal) per partner,
$m_{a,c\prime} = \frac{n_{a,x1,y1}(1-c_{x1,y1})(1-c_i)}{d_{x1,y1}}$ = number of annual unprotected sexual acts (anal) per partner,
$c_{x1,y1} = p_c c_c + p_{c+m} n_c c_c + p_{c+m} n_m c_m + p_m c_m$ = proportion of condom usage by risk group x_1, and age group y_1,
n_m = 1- n_c = proportion of acts with main partners,
$n_c = \frac{m_{c+m}S_c}{S_{acts}}$ = proportion of acts with casual partners,
(note: $\left((1-c_{x1,y1})c_i + c_{x1,y1}\right) + (1-c_{x1,y1})(1-c_i) = 1$ and $p_c + p_{c+m} + p_m = 1$);

$q_{x1,y1,i}$ is the number of infected partners from compartment i that a susceptible person in risk group x_1 and age group y_1 has, and is calculated as,

$$q_{x1,y1,i} = d_{x1,y1} \sum_{x2=1}^{3} \sum_{y2=13}^{100} risk_{x1,x2}\, age_{y1,y1} \frac{I_{x2,y2,i}}{N_{x2,y2}} \tag{4}$$

$d_{x1,y1}$ = number of partners for risk group x_1, and age group y_1 (calculated as weighted average of median number of partners for each partnership type and proportion of partnership type),
$risk_{x1,x2}$ = risk specific mixing proportion between risk group x_1 and x_2,
$age_{y1,y1}$ = age specific mixing proportion between age group y_1 and y_2,
$I_{x2,y2,i}$ = number of infected in risk group x_2, age group y_2, and infected compartment i, and
$N_{x2,y2}$ = number of people in risk group x_2, age group y_2.

Data related to the above parameters are presented in Tables A5 to A14.

The total number of new infections in the National-Model for all risk groups and age groups, can then be calculated as follows:

$$\sum_{x_1=1}^{3} \sum_{y_1=13}^{100} S_{x_1,y_1}\left(1 - \prod_{i=1}^{18}\{M_{x_1,y_1,i}\}^{q_{x1,y1,i}}\right) \tag{5}$$

Jurisdictional-Model

We estimate the number of new infections as in (2) but now also include jurisdictional mixing of sexual partnerships as follows.

Number of new infections in risk group x_1, age group y_1, and jurisdiction j =

$$S_{x_1,y_1,j}\left(1-\prod_{i=1}^{18}\{M_{x_1,y_1,i}\}^{\sum_{\hat{j}=j1}^{jn} mixing_{x_1,j,\hat{j}}\, q_{\hat{j},x_1,y_1,i}}\right), \quad (6)$$

where,
$S_{x_1,y_1,j}$ is the number of susceptible persons in risk group x_1, age group y_1, and jurisdiction j
$M_{x_1,y_1,i}$ is the same as in (3), and
$q_{\hat{j},x_1,y_1,i}$ is the number of infected partners from compartment i and jurisdiction $\hat{j}$ that a susceptible person in risk group x_1, age group y_1 and jurisdiction j has, and is calculated as,

$$q_{\hat{j},x_1,y_1,i} = d_{x1,y1}\sum_{x2=1}^{3}\sum_{y2=13}^{100}\left(risk_{x1,x2}age_{y1,y1}\frac{I_{x_2,y_2,i,\hat{j}}}{N_{x_2,y_2,\hat{j}}}\right) \quad (7)$$

$I_{x2,y2,i,\hat{j}}$ = number of infected in risk group x_2, age group y_2, compartment i, and jurisdiction $\hat{j}$,
$N_{x2,y2,\hat{j}}$ = number of people in risk group x_2, age group y_2, and jurisdiction $\hat{j}$,
$mixing_{x_1,j,\hat{j}}$ = proportion of mixing of risk group x_1 located in jurisdiction j with PWH located in jurisdiction $\hat{j}$, and
$j \; and \; \hat{j} \in \{j_1, j_2, \ldots, j_{96}\}$.

Table A5: Age group specific mixing of sexual partnerships* by risk group

Risk group	***Age group***	***13-17***	***18-24***	***25-29***	***30-24***	***35-39***	***40-44***	***45-64***	***65-100***
**HM**	13-17	91.1%	4.2%	1.1%	1.1%	1.1%	1.1%	0.2%	0.0%
	18-24	2.3%	92.1%	1.1%	1.1%	1.1%	1.1%	1.1%	0.0%
	25-29	6.8%	6.8%	82.0%	1.1%	1.1%	1.1%	1.1%	0.0%
	30-24	14.1%	14.1%	14.1%	54.1%	1.1%	1.1%	1.1%	0.0%
	35-39	5.4%	5.4%	5.4%	5.4%	76.2%	1.1%	1.1%	0.0%
	40-44	4.5%	4.5%	4.5%	4.5%	4.5%	76.2%	1.1%	0.0%
	45-64	3.9%	3.9%	3.9%	3.9%	3.9%	3.9%	76.2%	0.0%
	65-100	0.0%	0.0%	0.0%	0.0%	0.0%	0.0%	0.0%	0.0%
**HF**	13-17	91.1%	6.9%	0.5%	0.5%	0.5%	0.5%	0.0%	0.0%
	18-24	6.5%	91.1%	0.5%	0.5%	0.5%	0.5%	0.5%	0.0%
	25-29	0.5%	39.8%	57.7%	0.5%	0.5%	0.5%	0.5%	0.0%
	30-24	0.5%	43.0%	0.5%	54.5%	0.5%	0.5%	0.5%	0.0%
	35-39	1.5%	14.7%	0.5%	0.5%	81.8%	0.5%	0.5%	0.0%
	40-44	0.5%	15.7%	0.5%	0.5%	0.5%	81.8%	0.5%	0.0%
	45-64	0.0%	0.0%	0.0%	0.0%	0.0%	0.0%	100.0%	0.0%
	65-100	0.0%	0.0%	0.0%	0.0%	0.0%	0.0%	0.0%	0.0%
**MSM**	13-17	91.1%	4.7%	0.5%	0.1%	1.1%	0.8%	1.8%	0.0%
	18-24	4.8%	48.0%	4.8%	1.6%	3.8%	32.9%	4.1%	0.0%
	25-29	10.4%	16.4%	55.9%	13.5%	1.9%	1.5%	0.4%	0.0%
	30-24	0.2%	1.3%	37.6%	46.3%	2.7%	8.2%	3.7%	0.0%
	35-39	6.1%	24.6%	0.9%	5.3%	55.2%	1.3%	6.6%	0.0%
	40-44	3.4%	7.4%	10.5%	4.3%	9.3%	55.2%	9.9%	0.0%
	45-64	11.0%	10.4%	9.9%	10.5%	1.7%	1.2%	55.2%	0.0%
	65-100	0.0%	0.0%	0.0%	0.0%	0.0%	0.0%	0.0%	0.0%

* Diagonal estimates from [3] [19] and off diagonal elements were calibrated to match incidence by age groups
HM: heterosexual male; HF: heterosexual female; MSM: men who have sex with men

Table A6: Mixing of sexual partnership by risk group [3]

Risk group	*HM*	*HF*	*MSM*
**HM**	0	100.00%	0
**HF**	98.20%	0	1.80%
**MSM**	0	40%	60%

HM: heterosexual male; HF: heterosexual female; MSM: men who have sex with men

Table A7: Number of annual median partners by partnership type [23–25]

Risk group	*Casual-main*[*]	*Main only*	*Casual only*
**HM**	1	1	4
**HF**	1	1	4
**MSM**	2	1	5

HM: heterosexual male; HF: heterosexual female; MSM: men who have sex with men
[*] Main partners in casual and main relationship type

Table A8: Proportion of partnership type[*] by risk group [20–22]

Risk group	*Casual*	*Casual only*
**HM**	0.579	0.144
**HF**	0.579	0.144
**MSM**	0.652	0.307

HM: heterosexual male; HF: heterosexual female; MSM: men who have sex with men
[*] Proportion of main only partnerships = 1-proportion casual only partnerships and proportion of casual-main partnerships = proportion casual – proportion of casual only

Table A9: Proportion of condom usage[*] by risk group, partnership type and age group [28,29]

Age group	*HF-main*	*HF-casual*	*HM-main*	*HM-casual*	*MSM-main*	*MSM-casual*
**13-17**	51.3%	72.1%	76.5%	84.4%	28.1%	61.3%
**18-24**	26.9%	41.7%	23.1%	48.9%	28.1%	61.3%
**25-29**	18.4%	39.3%	18.4%	49.6%	25.0%	54.5%
**30-39**	12.4%	24.9%	14.2%	48.9%	22.2%	48.4%
**40-49**	10.1%	18.4%	12.6%	30.6%	21.7%	47.3%
**50-59**	7.0%	14.9%	1.6%	20.8%	21.3%	46.6%
**60-100**	3.8%	17.4%	2.0%	12.5%	20.1%	43.8%

HM: heterosexual male; HF: heterosexual female; MSM: men who have sex with men
[*] Condom efficiency is assumed to be 80% [30–32]

Table A10: Annual sexual acts[*] by age group and risk group

Age group	*HM*	*HF*	*MSM*
**13-14**	45	24	45
**15-17**	45	24	45
**18-19**	84	94	107
**20-24**	84	94	107
**25-29**	81	78	99
**30-34**	73	66	93
**35-39**	73	66	93
**40-44**	70	67	82
**45-49**	77	67	61
**50-54**	54	59	56
**55-59**	54	47	56
**60-64**	55	48	37
**65-70**	55	48	37

HM: heterosexual male; HF: heterosexual female; MSM: men who have sex with men
[*] Ranges for sexual acts from [24–27]

Table A11: Proportion of annual sexual acts*, by risk group and age group [24–27]

Age group	*HF*	*HM*	*MSM*
**13-24**	6.6%	4.8%	50%
**25-29**	7.5%	8.4%	50%
**30-39**	5.9%	4.2%	50%
**40-49**	3.9%	6.1%	50%
**50-59**	2.5%	2.8%	50%
**60-100**	4.2%	3.7%	50%

HM: heterosexual male; HF: heterosexual female; MSM: men who have sex with men
* Proportion of vaginal acts = 1-proportion of anal acts

Table A12: Scalar risk factor* for transmission in various stages of care and disease [15,33–36]

Compartment	*Transmission risk scalar factor*
Acute stages	8.1
Non-acute with viral load suppression	0.01
Non-acute without viral load suppression	1

* Usage of PrEP reduces transmission risk by 99% [37]

Table A13: Calibrated values of probability of HIV transmission* per sexual act for risk groups

Risk group	*Vaginal acts*	*Anal acts*
**HM**	0.0007	0.00160
**HF**	0.0004	0.00831
**MSM**	0.0018	0.00586

HM: heterosexual male; HF: heterosexual female; MSM: men who have sex with men
* Initial estimates and ranges for transmission probability were taken from [38–40]

Table A14: Proportion of men who have sex with men* among male population by county [41]

County	*Proportion*
Maricopa County	0.06
Alameda County	0.07
Los Angeles County	0.07
Orange County	0.06
Riverside County	0.09
Sacramento County	0.07
San Bernardino County	0.03
San Diego County	0.07
Broward County	0.09
Duval County	0.05
Hillsborough County	0.06
Miami-Dade County	0.06
Orange County	0.07
Palm Beach County	0.05
Pinellas County	0.07
Cobb County	0.04
Dekalb County	0.08
Fulton County	0.09
Gwinnett County	0.04
Cook County	0.07
Marion County	0.06
East Baton Rouge Parish	0.03

Orleans Parish	0.03
Baltimore City	0.04
Montgomery County	0.04
Prince George's County	0.04
Wayne County	0.05
Mecklenburg County	0.06
Essex County	0.04
Hudson County	0.07
Clark County	0.06
Bronx County	0.05
Kings County	0.07
New York County	0.14
Queens County	0.05
Cuyahoga County	0.06
Franklin County	0.07
Hamilton County	0.03
Philadelphia County	0.06
Shelby County	0.05
Bexar County	0.05
Dallas County	0.08
Harris County	0.06
Tarrant County	0.05
Travis County	0.08
King County	0.08
National	0.04

* Data for some counties is not available in [41], for such counties, we use the same percentage as the state. For states that have multiple EHE counties, MSM population in counties was removed to calculate the proportion of MSM population within a state.

A4: Estimation of diagnosis and retention-in-care rates

As care parameters change over time, the diagnosis and retention in care rates also change. Therefore, we analytically estimate these in the model, by calibrating it to the expected targets for the care continuum metrics, specifically, the % aware, and % VLS. We calculate diagnosis rate and retention-in-care rate specific to risk group and jurisdiction only (and not specific to age or disease stage), and thus use a collapsed/simplified state of the Markov process (Eqn. 1 in Section A1) as follows (see Figure A1).

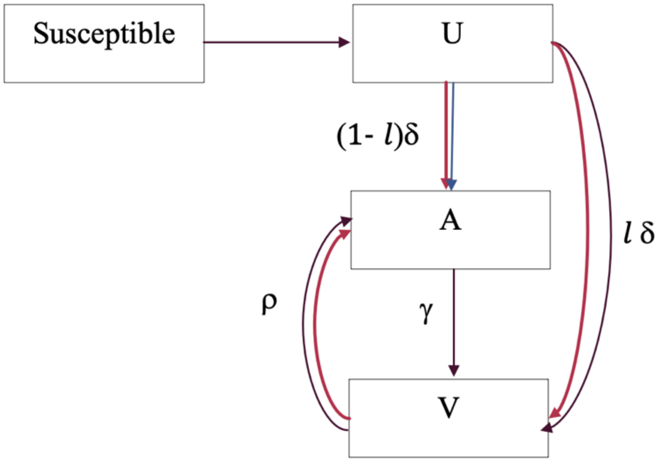

Figure A1: *Flow diagram for disease incidence and transition[*] along the stages of care continuum[**]*

[*] ***δ****: diagnosis rate,* ***γ****: rate of entering care and treatment among those not in care, and* ***ρ****: rate of dropping out of care, and* ***l****: proportion linked-to-care at diagnosis*
[**] *Susceptible: Population susceptible, U: population Unaware, A: population Aware no ART, V: population with ART no VLS and ART VLS*

For a sufficiently small incremental time-step, $t+1$ (we use monthly increments), we can write the generalized compartmental model for the number of people in each stage by formulating it as a system of differential equations.

$$p_{t+1,s,risk}I_{t+1} = p_{t,s,risk}I_t + \frac{dp_{t,s,risk}I_t}{dt}, \quad (8)$$

Where,

$p_{t,s,risk}$ is the proportion of people in care continuum stage s; at time t for risk group $risk$,
I_t = number of PWH at time t (estimated prevalence),
$\frac{dp_{t,s,risk}I_t}{dt}$ is the rate of change in $p_{t,s,risk}I_t$, i.e., the change in the number of infected persons in stage s at time t,
dt = 1/12 (monthly),
s = care continuum stage; $s \in \{U, A, V\}$; U = unaware; A= aware; V = prescribed ART (with VLS + no VLS).

We estimate rates δ_t and ρ_t by expansion of the above equations as discussed in following sub-sections. We estimate these rates specific to risk group for the National-Model and specific to both risk group and jurisdiction for the Jurisdictional-Model but exclude the jurisdictional notation for clarity.

A4.1 Estimation of diagnosis rates

Expanding (8) for $s = U$ (Unaware stage), we can write,

$$I_t p_{t,U,risk} = I_{t-1}p_{t-1,U,risk} + i_{t,risk} - \delta_{t,risk}\sum_d \theta_{d,risk} I_{t-1,} p_{t-1,U,risk,d} - \sum_d m_{t,U,risk} \quad (9)$$

Where,

I_t = total number of people living with HIV (PWH) (estimated prevalence),
$p_{t,U,risk}$ is the proportion of people in care continuum stage U (here $s = \{U\}$) for risk group $risk$ at time t,
$i_{t,risk}$= new infections generated at time t in risk group $risk$,
$\delta_{t,risk}$ = diagnosis rate at time t for each risk group $risk$,
$\theta_{d,risk}$= scaling factor for diagnosis rate in disease-stage d for each risk group $risk$ (see footnotes for Table A2); $d \in \{Acute, CD4 > 500, CD4\ 350-500, CD4\ 200-350, CD4 < 200\}$,
$p_{t,U,risk,d}$ is the proportion of people in care continuum stage U (here $s = \{U\}$) for risk group $risk$ at time t and for disease stage d, and
$m_{t,U,d,risk}$ = number of deaths in the care-stage U (here $s = \{U\}$) and disease-stage d at time t in each risk group $risk$.

Rearranging (9) we can solve for diagnostic rate $\delta_{t,risk}$ as

$$\delta_{t,risk} = \frac{i_{t,risk}+I_{t-1}\,p_{t-1,U,risk}-I_t\,p_{t,U,risk}-\sum_d(m_{t,U,d,risk})}{\sum_d \theta_d I_{t-1}p_{t-1,U,risk,d}} \quad (10)$$

and the corresponding number of people that are diagnosed as $\delta_{t,risk}\sum_d \theta_d\, I_{t-1}p_{t-1,U,risk,d}$

Each term on the right-hand-side of (10) is computationally calculated in the simulation as follows:

- $i_{t,risk}$ is number of new infection for each risk group and is calculated using the Bernoulli equations (Section A2),
- $\sum_d(m_{t,U,d,risk})$ is the number of deaths andtracked in the simulation (death rates presented in Tables A3 and A4),
- $I_{t-1}\,p_{t-1,U,risk}$ is the number of people in compartment U at previous time-step and is tracked in the simulation (initial data for distribution of population in care stages, i.e., $p_{t,s,risk}$ for both National-Model

and Jurisdictional-Model, are taken from NHSS data [16] and projections over time are tracked in the simulation),

- $p_{t,U,risk}$ is the expected proportion of people in compartment U at time-step t for each risk group,
- $I_t\, p_{t,U,risk}$ is the expected number of people in compartment U in time-step t to match the expected value of $p_{t,U,risk}$ and is calculated as $p_{t,U,risk} = p_{t-1,U,risk} + \frac{a_{U,T,risk} - a_{U,T-1,risk}}{1/dt}$,
  - $a_{U,T-1,risk}$ is the proportion of people unaware in previous year T-1 and risk group $risk$,
  - $a_{U,T,risk}$ is the proportion of people unaware in year T and risk group $risk$ (for baseline scenarios, proportion unaware is the actual value in the U.S. in year 2018; for EHE plan scenarios, proportion unaware is scaled up every year from current value in 2018 to reach EHE target of 5% unaware by 2025 for EHE jurisdictions and by 2030 for non-EHE jurisdictions), and
  - $\frac{a_{U,T,risk} - a_{U,T-1,risk}}{1/dt}$ is the expected change in proportion of persons unaware of infection.

A4.2 Estimation of dropout rates

We are only estimating the dropout rate for CD4 count >200. For CD4 count <200, we assume dropout is 0 and this is modeled by making φ_d = 0 for CD4 count < 200.

Expanding (8) for $s = V$ (prescribed ART) we can write,

$$I_t p_{t,V,risk} = I_{t-1}p_{t-1,V,risk} + \delta_{t,risk} l_{t,risk} \sum_d I_{t-1}p_{t-1,U,d,risk}\theta_{d,risk} + \sum_d \gamma_d I_{t-1}p_{t-1,A,d,risk} - \rho_{t,risk}\sum_d I_{t-1}p_{t-1,V,d,risk}\varphi_d - \sum_d\left(m_{t,V,d,risk}\right) \quad (11)$$

Where,

I_t is the total number of people living with HIV (PWH) (estimated prevalence),
$p_{t,V,risk}$ is the proportion of people in care continuum stage V (here $s = \{V\}$) for risk group $risk$ at time t,
$\delta_{t,risk}$ is the diagnosis rate at time t for each risk group $risk$ (as estimated in section A3.1),
$l_{t,risk}$ is the proportion linked-to-care at diagnosis at time t for each risk group $risk$ (data for both National-Model and Jurisdictional-Model are taken from NHSS data [16]),
$\theta_{d,risk}$ is the scaling factor for diagnosis rate in disease-stage d for each risk group $risk$ (see footnotes for Table A2); $d \in \{Acute, CD4 > 500, CD4\ 350-500, CD4\ 200-350, CD4 < 200\}$,
γ_d is the re-entry rate for disease stage d (assumed 0.5 per year for CD4 >= 200 and 1 per year for CD4 < 200 [9]),
$p_{t-1,A,d,risk}$ is the proportion of people in care continuum stage A (here $s = \{A\}$) for risk group $risk$ at time $t-1$,
$\rho_{t,risk}$ is the dropout rate at time t (dropout rate for CD4 < 200 = 0, because <200 is opportunistic infection/AIDS so we assume they will stay in care) for each risk group $risk$,
φ_d is the scaling factor for drop-out rate in disease-stage d (see footnotes for Table A2), and
$m_{t,V,d,risk}$ is the number of deaths in the care-stage V (here $s = \{V\}$) and disease-stage d at time t in each risk group $risk$.

Rearranging (11) we can solve for dropout rate $\rho_{t,risk}$ as

$$\rho_{t,risk} = \frac{I_{t-1}p_{t-1,V,risk} + \delta_{t,risk} l_{t,risk}\sum_d I_{t-1}p_{t-1,U,d,risk}\theta_d + \sum_d \gamma_d I_{t-1}p_{t-1,A,d,risk} - \sum_d\left(m_{t,V,d,risk}\right) - I_t p_{t,V,risk}}{\sum_d I_{t-1}p_{t-1,V,d,risk}\varphi_d} \quad (12)$$

and the corresponding number of people that drop out of care as $\rho_{t,risk}\sum_d \varphi_d\, I_{t-1}p_{t-1,V,risk,d}$

Each term in the right-hand-side of (12) is computationally calculated in the simulation as follows:

- $I_{t-1}\, p_{t-1,V,risk}$ is the number of people in compartment V at previous time-step and is tracked in the simulation (initial data for distribution of population in care stages, i.e., $p_{t,s,risk}$ for both National-Model and Jurisdictional-Model, are taken from NHSS data [16] and projections over time are tracked in the simulation),

- $\delta_{t,risk} l_{t,risk} \sum_d I_{t-1} p_{t-1,U,d,risk} \theta_d$ is the number of people linked-to-care at diagnosis and is calculated from estimation of diagnosis rates (section A3.1) and tracked in the simulation,
- $\sum_d \gamma_d I_{t-1} p_{t-1,A,d,risk}$ is the number of people who enter care in the previous time step and is tracked in the simulation,
- $\sum_d (m_{t,V,d,risk})$ is the number of deaths and is tracked in the simulation (death rates presented in Tables A3 and A4),
- $p_{t,V,risk}$ is the expected proportion of people in compartment V at time-step t for each risk group,
- $I_t\, p_{t,V,risk}$ is the expected number of people in compartment V in time-step t to match the expected value of $p_{t,V,risk}$, which we can calculate as $p_{t,V,risk} = p_{t-1,V,risk} + \frac{a_{V,T,risk} - a_{V,T-1,risk}}{1/dt}$,
    - $a_{V,T-1,risk}$ is the proportion of people on ART (with and without VLS) for previous year T-1 and risk group $risk$,
    - $a_{V,T,risk}$ is the proportion of people on ART (with and without VLS) for year T and risk group $risk$ (for baseline scenarios, proportion ART (with and without VLS) is the actual value in the U.S. in year 2018; for EHE plan scenarios, proportion ART (with and without VLS) is scaled up every year from current value in 2019 to reach EHE target of 85.7% (calculated as 0.95*0.95*0.95, as per the 95-95-95 care continuum targets of reach 95% aware, 95% linkage-to-care among aware, and 95% VLS among those in care) by 2025 for EHE jurisdictions and by 2030 for non-EHE jurisdictions, and
    - $\frac{a_{V,T,risk} - a_{V,T-1,risk}}{1/dt}$ is the expected change in proportion of persons on ART (with and without VLS).

A5: Estimation of jurisdiction-specific proportion aware categorized by risk group

Data on jurisdiction-specific care continuum distributions categorized by risk group are not available. Therefore, we made approximate estimations as discussed below for proportion aware. We use similar calculations for proportion Aware no ART, and ART (which combines ART no VLS and ART VLS).

Let,
$unaware_{j,r}$= proportion of people unaware in jurisdiction j, for risk group r,
$unaware_j$ = proportion of people unaware in jurisdiction j,
$unaware_{nat,r}$ = proportion of people unaware in national data, for risk group r,
$unaware_{nat}$ = proportion of people unaware in national data,
$r \in \{Heterosexual\ males, Heterosexual\ females, Men\ who\ have\ sex\ with\ men\}$, and
$j \in \{jur_1, jur_2, \ldots, jur_{96}\}$.

Then we can write,

$$\frac{unaware_{j,r}}{unaware_j} = \frac{unaware_{nat,r}}{unaware_{nat}} \quad (5)$$

$$unaware_{j,r} = \frac{unaware_{nat,r}}{unaware_{nat}}\, unaware_j \quad (6)$$

we can calculate $unaware_{j,r}$ from the equation (4).

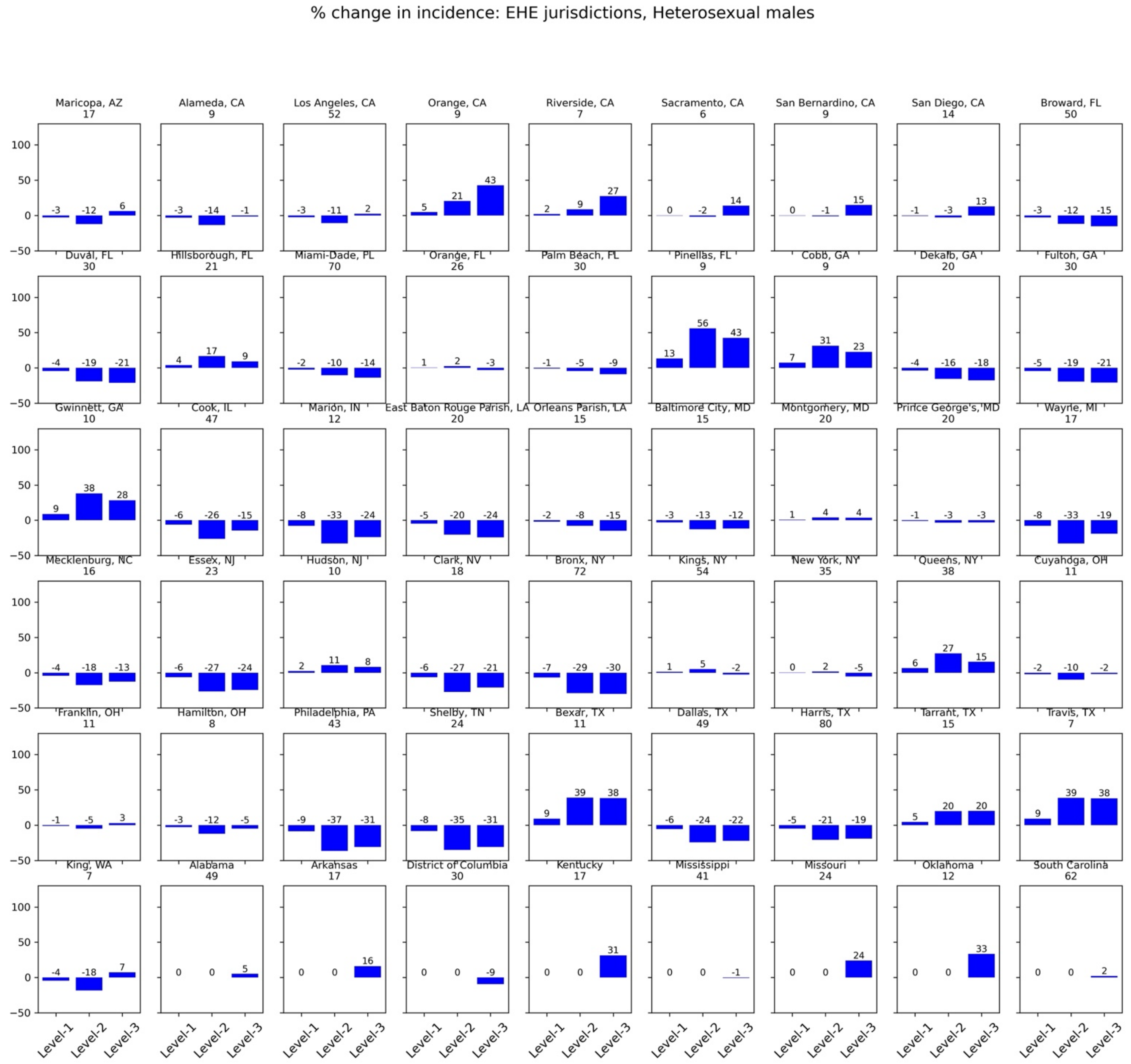

Figure A2a: Percentage change in incidence in mixing scenario compared to no-mixing (Heterosexual males, EHE jurisdictions*, baseline intervention, 2018)

Level-1: Scenario S14; Level-2: Scenario S15; and Level-3: Scenario S16

* The title on each subplot is the EHE jurisdiction (county or state) along with values of incidence in year 2018 under the no-mixing scenario [S13]

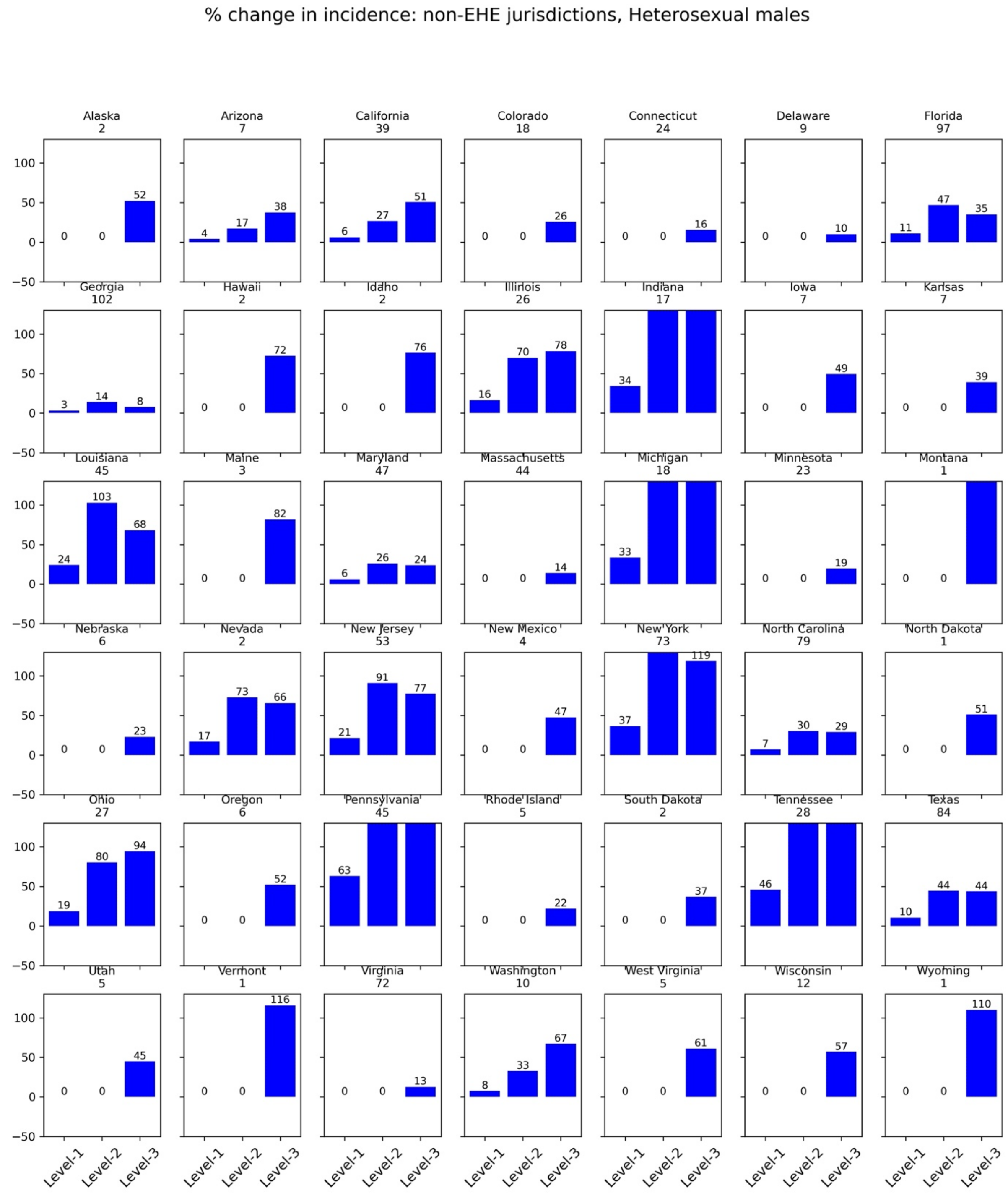

Figure A2b: Percentage change in incidence in mixing scenario compared to no-mixing (Heterosexual males, non-EHE jurisdictions*, baseline intervention, 2018)

Level-1: Scenario S14; Level-2: Scenario S15; and Level-3: Scenario S16

* The title on each subplot is the non-EHE jurisdiction (state) along with values of incidence in year 2018 under the no-mixing scenario [S13]

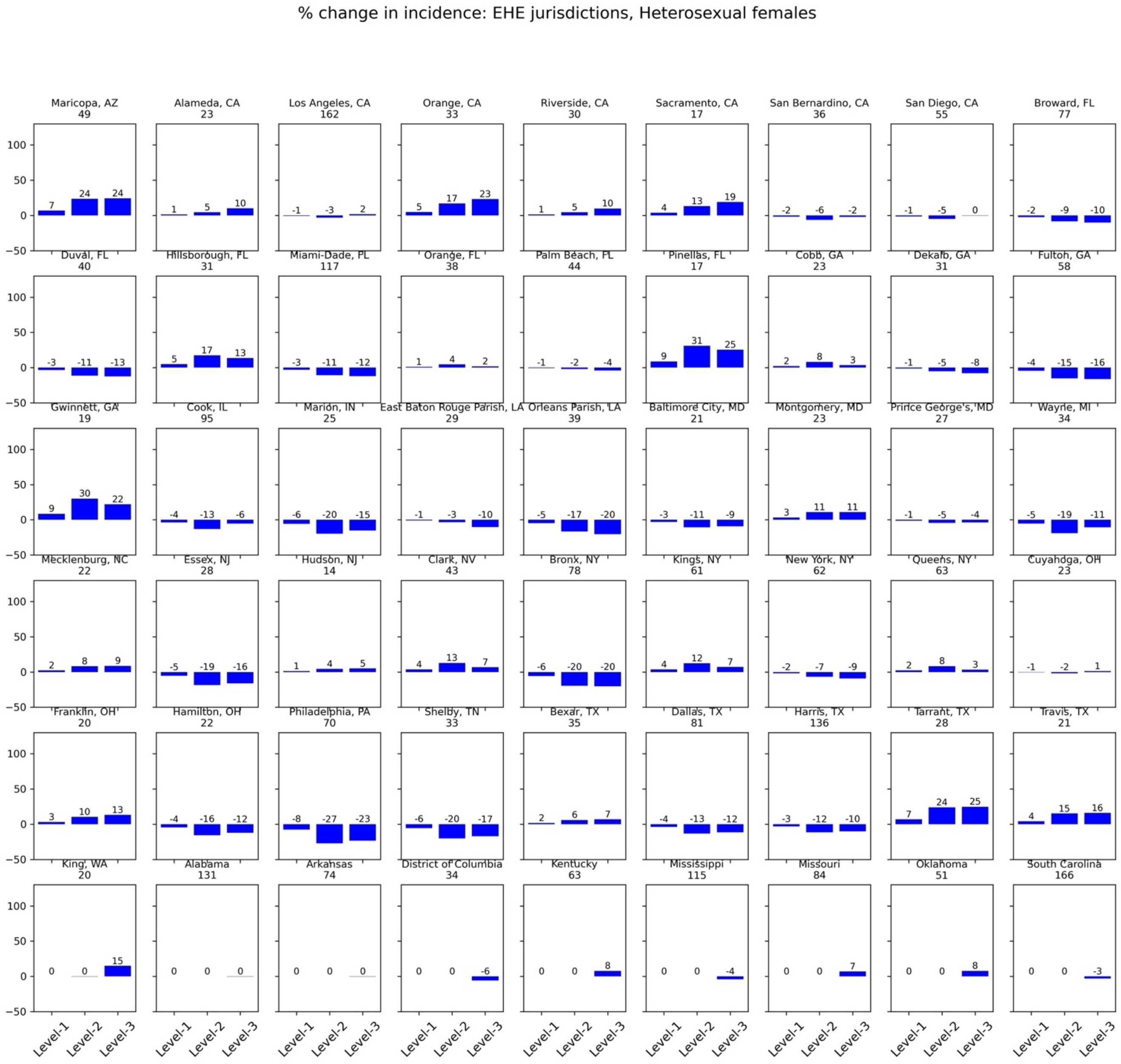

Figure A3a: Percentage change in incidence in mixing scenario compared to no-mixing (Heterosexual females, EHE jurisdictions*, baseline intervention, 2018)

Level-1: Scenario S14; Level-2: Scenario S15; and Level-3: Scenario S16

* The title on each subplot is the EHE jurisdiction (county or state) along with values of incidence in year 2018 under the no-mixing scenario [S13]

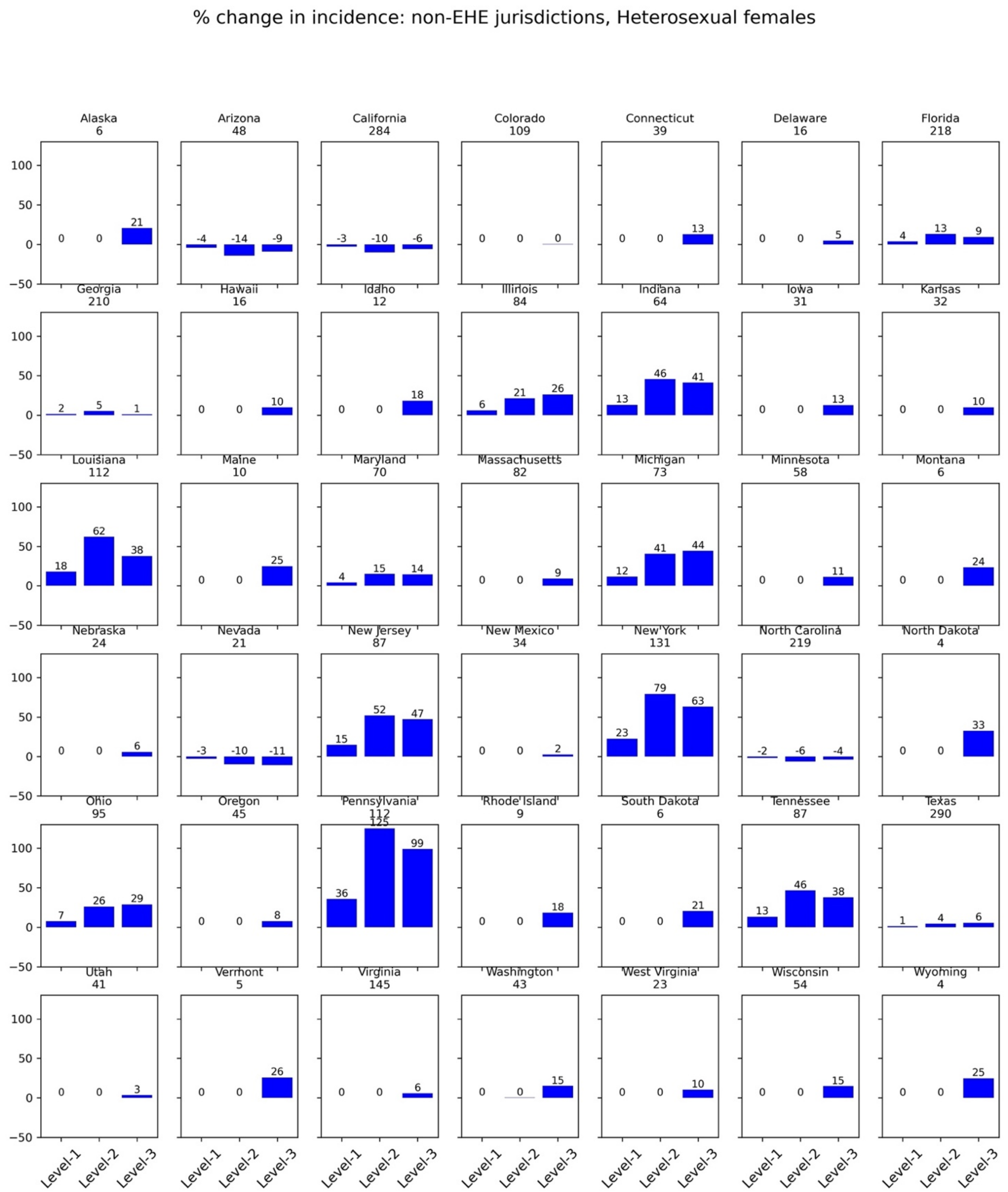

Figure A3b: Percentage change in incidence in mixing scenario compared to no-mixing (Heterosexual females, non-EHE jurisdictions*, baseline intervention, 2018)

Level-1: Scenario S14; Level-2: Scenario S15; and Level-3: Scenario S16

* The title on each subplot is the non-EHE jurisdiction (state) along with values of incidence in year 2018 under the no-mixing scenario [S13]

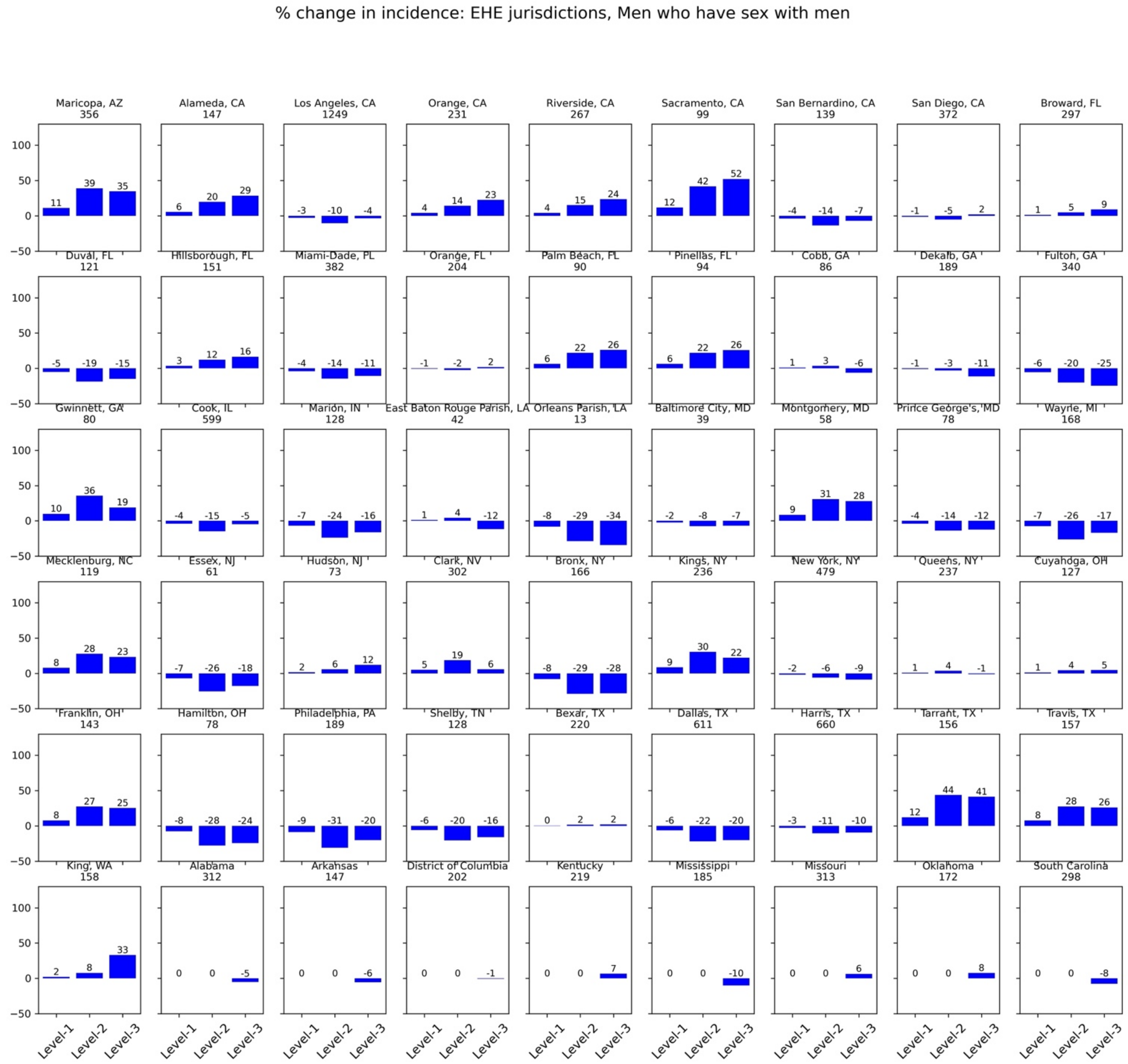

Figure A4a: Percentage change in incidence in mixing scenario compared to no-mixing (Men who have sex with men, EHE jurisdictions*, baseline intervention, 2018)

Level-1: Scenario S14; Level-2: Scenario S15; and Level-3: Scenario S16
* The title on each subplot is the EHE jurisdiction (county or state) along with values of incidence in year 2018 under the no-mixing scenario [S13]

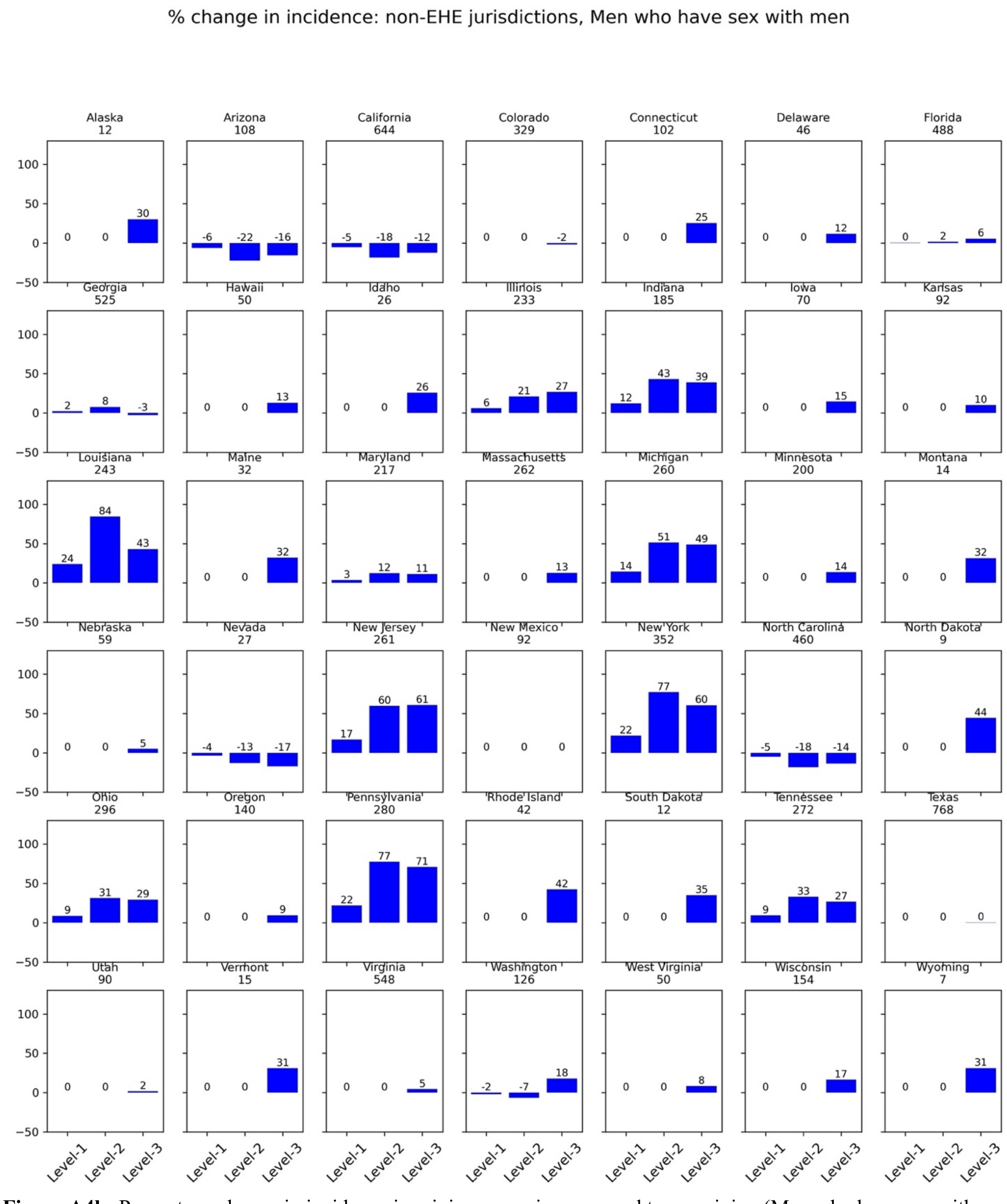

Figure A4b: Percentage change in incidence in mixing scenario compared to no-mixing (Men who have sex with men, non-EHE jurisdictions*, baseline intervention, 2018)

Level-1: Scenario S14; Level-2: Scenario S15; and Level-3: Scenario S16

* The title on each subplot is the non-EHE jurisdiction (state) along with values of incidence in year 2018 under the no-mixing scenario [S13]

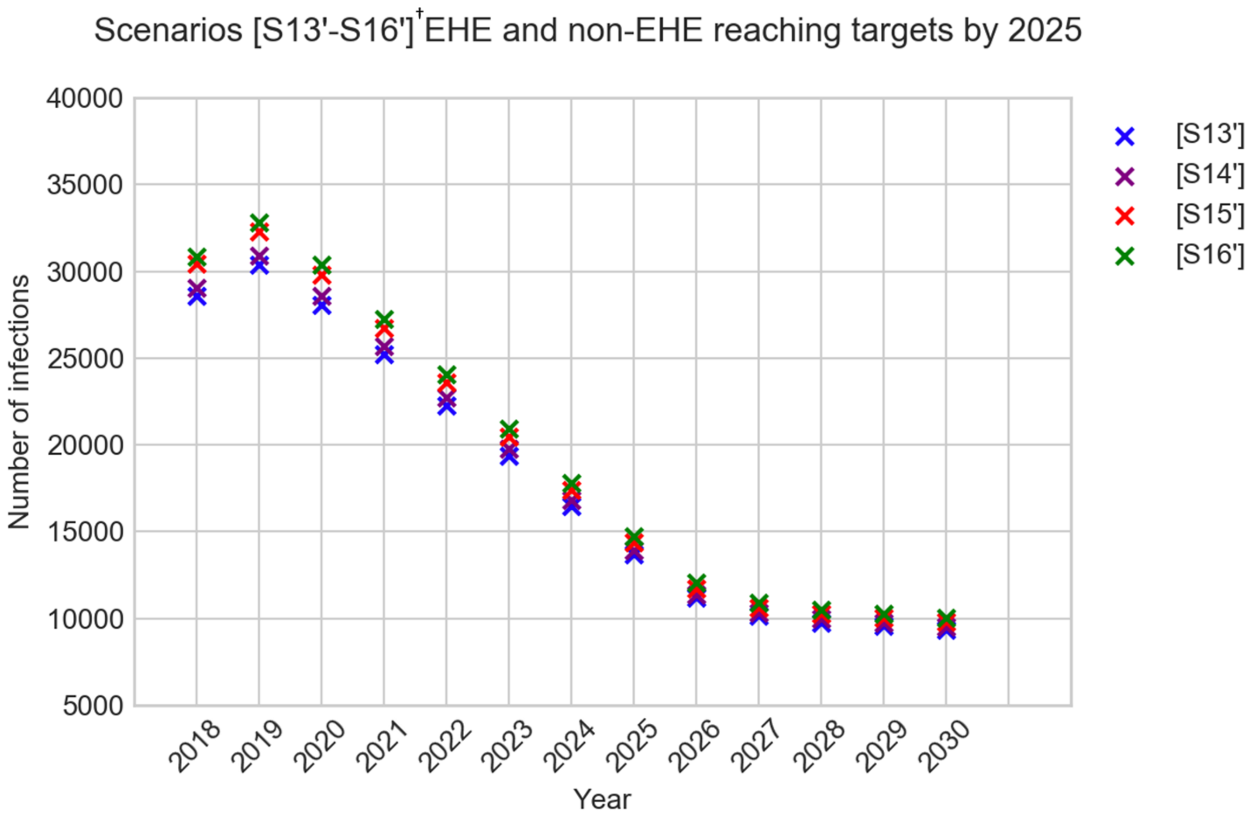

Figure A5: Comparing annual incidence projections of modified EHE-plan-intervention†; jurisdiction-heterogeneity in care scenarios

† Scenarios S13, S14, S15, and S16, implement the EHE plan where EHE jurisdiction reach EHE targets by 2025 and non-EHE jurisdictions reach EHE targets by 2030. Modified scenario S13', S14', S15', and S16', implement the EHE plan where both, EHE and non-EHE jurisdiction, reach EHE targets by 2025.

References

1. CDC. Ending the HIV Epidemic in the U.S. (EHE). 7 Sep 2021 [cited 14 Dec 2021]. Available: https://www.cdc.gov/endhiv/jurisdictions.html?CDC_AA_refVal=https%3A%2F%2Fwww.cdc.gov%2Fendhiv%2Fpriorities.html

2. Social Security. Actuarial Life Table. [cited 9 Nov 2021]. Available: https://www.ssa.gov/oact/STATS/table4c6.html

3. Khurana N, Yaylali E, Farnham PG, Hicks KA, Allaire BT, Jacobson E, et al. Impact of Improved HIV Care and Treatment on PrEP Effectiveness in the United States, 2016–2020. JAIDS Journal of Acquired Immune Deficiency Syndromes. 2018;78: 399–405. doi:10.1097/QAI.0000000000001707

4. Fiebig EW, Wright DJ, Rawal BD, Garrett PE, Schumacher RT, Peddada L, et al. Dynamics of HIV viremia and antibody seroconversion in plasma donors: implications for diagnosis and staging of primary HIV infection. AIDS. 2003;17: 1871–1879. doi:10.1097/00002030-200309050-00005

5. Importance of Baseline Prognostic Factors With Increasing Time Since Initiation of Highly Active Antiretroviral Therapy: Collaborative Analysis of Cohorts of HIV-1-Infected Patients. JAIDS Journal of Acquired Immune Deficiency Syndromes. 2007;46: 607–615. doi:10.1097/QAI.0b013e31815b7dba

6. Mellors JW. Plasma Viral Load and CD4+ Lymphocytes as Prognostic Markers of HIV-1 Infection. Ann Intern Med. 1997;126: 946. doi:10.7326/0003-4819-126-12-199706150-00003

7. Time from HIV-1 seroconversion to AIDS and death before widespread use of highly-active antiretroviral therapy: a collaborative re-analysis. The Lancet. 2000;355: 1131–1137. doi:10.1016/S0140-6736(00)02061-4

8. Dorrucci M, Rezza G, Porter K, Phillips A, Concerted Action on Seroconversion to AIDS and Death in Europe Collaboration. Temporal Trends in Postseroconversion CD4 Cell Count and HIV Load: The Concerted Action on Seroconversion to AIDS and Death in Europe Collaboration, 1985–2002. J INFECT DIS. 2007;195: 525–534. doi:10.1086/510911

9. Gardner EM, McLees MP, Steiner JF, del Rio C, Burman WJ. The Spectrum of Engagement in HIV Care and its Relevance to Test-and-Treat Strategies for Prevention of HIV Infection. Clinical Infectious Diseases. 2011;52: 793–800. doi:10.1093/cid/ciq243

10. Clinical Info HIV.gov. Guidelines for the Use of Antiretroviral Agents in Adults and Adolescents Living with HIV. 18 Dec 2019 [cited 9 Nov 2021]. Available: https://clinicalinfo.hiv.gov/en/guidelines/adult-and-adolescent-arv/initiation-antiretroviral-therapy

11. Long EF, Brandeau ML, Owens DK. Potential population health outcomes and expenditures of HIV vaccination strategies in the United States. Vaccine. 2009;27: 5402–5410. doi:10.1016/j.vaccine.2009.06.063

12. Dunn D, Woodburn P, Duong T, Peto J, Phillips A, Gibb D, et al. Current CD4 Cell Count and the Short-Term Risk of AIDS and Death before the Availability of Effective Antiretroviral Therapy in HIV-Infected Children and Adults. J INFECT DIS. 2008;197: 398–404. doi:10.1086/524686

13. Long EF, Brandeau ML, Owens DK. The Cost-Effectiveness and Population Outcomes of Expanded HIV Screening and Antiretroviral Treatment in the United States. Ann Intern Med. 2010;153: 778. doi:10.7326/0003-4819-153-12-201012210-00004

14. Juusola JL, Brandeau ML, Owens DK, Bendavid E. The Cost-Effectiveness of Preexposure Prophylaxis for HIV Prevention in the United States in Men Who Have Sex With Men. Ann Intern Med. 2012;156: 541. doi:10.7326/0003-4819-156-8-201204170-00001

15. Gopalappa C, Farnham PG, Chen Y-H, Sansom SL. Progression and Transmission of HIV/AIDS (PATH 2.0): A New, Agent-Based Model to Estimate HIV Transmissions in the United States. Med Decis Making. 2017;37: 224–233. doi:10.1177/0272989X16668509

16. NCHHSTP AtlasPlus. [cited 4 Nov 2021]. Available: https://www.cdc.gov/nchhstp/atlas/index.htm

17. Grover D, Copas A, Green H, Edwards SG, Dunn DT, Sabin C, et al. What is the risk of mortality following diagnosis of multidrug-resistant HIV-1? Journal of Antimicrobial Chemotherapy. 2008;61: 705–713. doi:10.1093/jac/dkm522

18. Prognosis of HIV-1-infected patients up to 5 years after initiation of HAART: collaborative analysis of prospective studies. AIDS. 2007;21: 1185–1197. doi:10.1097/QAD.0b013e328133f285

19. Glick SN, Morris M, Foxman B, Aral SO, Manhart LE, Holmes KK, et al. A Comparison of Sexual Behavior Patterns Among Men Who Have Sex With Men and Heterosexual Men and Women. JAIDS Journal of Acquired Immune Deficiency Syndromes. 2012;60: 83–90. doi:10.1097/QAI.0b013e318247925e

20. Rosenberg ES, Sullivan PS, DiNenno EA, Salazar LF, Sanchez TH. Number of casual male sexual partners and associated factors among men who have sex with men: Results from the National HIV Behavioral Surveillance system. BMC Public Health. 2011;11: 189. doi:10.1186/1471-2458-11-189

21. Voetsch AC, Lansky A, Drake AJ, MacKellar D, Bingham TA, Oster AM, et al. Comparison of Demographic and Behavioral Characteristics of Men Who Have Sex With Men by Enrollment Venue Type in the National HIV Behavioral Surveillance System. Sexually Transmitted Diseases. 2012;39: 229–235. doi:10.1097/OLQ.0b013e31823d2b24

22. Finlayson TJ, Le B, Smith A, Bowles K, Cribbin M, Miles I, et al. HIV risk, prevention, and testing behaviors among men who have sex with men--National HIV Behavioral Surveillance System, 21 U.S. cities, United States, 2008. MMWR Surveill Summ. 2011;60: 1–34.

23. Chandra A, Copen CE, Mosher WD. Sexual Behavior, Sexual Attraction, and Sexual Identity in the United States: Data from the 2006–2010 National Survey of Family Growth. In: Baumle AK, editor. International Handbook on the Demography of Sexuality. Dordrecht: Springer Netherlands; 2013. pp. 45–66. doi:10.1007/978-94-007-5512-3_4

24. Reece M, Herbenick D, Schick V, Sanders SA, Dodge B, Fortenberry JD. Sexual Behaviors, Relationships, and Perceived Health Among Adult Men in the United States: Results from a National Probability Sample. The Journal of Sexual Medicine. 2010;7: 291–304. doi:10.1111/j.1743-6109.2010.02009.x

25. Herbenick D, Reece M, Schick V, Sanders SA, Dodge B, Fortenberry JD. Sexual Behaviors, Relationships, and Perceived Health Status Among Adult Women in the United States: Results from a National Probability Sample. The Journal of Sexual Medicine. 2010;7: 277–290. doi:10.1111/j.1743-6109.2010.02010.x

26. Reece M, Herbenick D, Schick V, Sanders SA, Dodge B, Fortenberry JD. Background and Considerations on the National Survey of Sexual Health and Behavior (NSSHB) from the Investigators. The Journal of Sexual Medicine. 2010;7: 243–245. doi:10.1111/j.1743-6109.2010.02038.x

27. Herbenick D, Reece M, Schick V, Sanders SA, Dodge B, Fortenberry JD. Sexual Behavior in the United States: Results from a National Probability Sample of Men and Women Ages 14–94. The Journal of Sexual Medicine. 2010;7: 255–265. doi:10.1111/j.1743-6109.2010.02012.x

28. Reece M, Herbenick D, Schick V, Sanders SA, Dodge B, Fortenberry JD. Condom Use Rates in a National Probability Sample of Males and Females Ages 14 to 94 in the United States. The Journal of Sexual Medicine. 2010;7: 266–276. doi:10.1111/j.1743-6109.2010.02017.x

29. Rosenberger JG, Reece M, Schick V, Herbenick D, Novak DS, Van Der Pol B, et al. Condom Use during Most Recent Anal Intercourse Event among a U.S. Sample of Men Who Have Sex with Men. The Journal of Sexual Medicine. 2012;9: 1037–1047. doi:10.1111/j.1743-6109.2012.02650.x

30. Pinkerton SD, Abramson PR. Effectiveness of condoms in preventing HIV transmission. Social Science & Medicine. 1997;44: 1303–1312. doi:10.1016/S0277-9536(96)00258-4

31. Weller SC, Davis-Beaty K. Condom effectiveness in reducing heterosexual HIV transmission. Cochrane HIV/AIDS Group, editor. Cochrane Database of Systematic Reviews. 2002;2012. doi:10.1002/14651858.CD003255

32. Davis KR, Weller SC. The effectiveness of condoms in reducing heterosexual transmission of HIV. Fam Plann Perspect. 1999;31: 272–279.

33. Hughes JP, Baeten JM, Lingappa JR, Magaret AS, Wald A, de Bruyn G, et al. Determinants of Per-Coital-Act HIV-1 Infectivity Among African HIV-1–Serodiscordant Couples. The Journal of Infectious Diseases. 2012;205: 358–365. doi:10.1093/infdis/jir747

34. Hollingsworth TD, Anderson RM, Fraser C. HIV-1 Transmission, by Stage of Infection. J INFECT DIS. 2008;198: 687–693. doi:10.1086/590501

35. Pilcher CD, Tien HC, Eron, Jr. JJ, Vernazza PL, Leu S, Stewart PW, et al. Brief but Efficient: Acute HIV Infection and the Sexual Transmission of HIV. J INFECT DIS. 2004;189: 1785–1792. doi:10.1086/386333

36. Pilcher CD, Joaki G, Hoffman IF, Martinson FE, Mapanje C, Stewart PW, et al. Amplified transmission of HIV-1: comparison of HIV-1 concentrations in semen and blood during acute and chronic infection. AIDS. 2007;21: 1723–1730. doi:10.1097/QAD.0b013e3281532c82

37. CDC. PrEP Effectiveness. 13 May 2021 [cited 20 Aug 2021]. Available: https://www.cdc.gov/hiv/basics/prep/prep-effectiveness.html

38. Patel P, Borkowf CB, Brooks JT, Lasry A, Lansky A, Mermin J. Estimating per-act HIV transmission risk: a systematic review. AIDS. 2014;28: 1509–1519. doi:10.1097/QAD.0000000000000298

39. Pretty IA, Anderson GS, Sweet DJ. Human Bites and the Risk of Human Immunodeficiency Virus Transmission: The American Journal of Forensic Medicine and Pathology. 1999;20: 232–239. doi:10.1097/00000433-199909000-00003

40. Pebody R. Estimated HIV risk per exposure. In: aidsmap: The biology of HIV transmission [Internet]. May 2020. Available: https://www.aidsmap.com/about-hiv/estimated-hiv-risk-exposure

41. Grey JA, Bernstein KT, Sullivan PS, Purcell DW, Chesson HW, Gift TL, et al. Estimating the Population Sizes of Men Who Have Sex With Men in US States and Counties Using Data From the American Community Survey. JMIR Public Health Surveill. 2016;2: e14. doi:10.2196/publichealth.5365

42. Board AR, Oster AM, Song R, Gant Z, Linley L, Watson M, et al. Geographic Distribution of HIV Transmission Networks in the United States. JAIDS Journal of Acquired Immune Deficiency Syndromes. 2020;85: e32–e40. doi:10.1097/QAI.0000000000002448
